# RAB7 protects against ischemic heart failure via promoting non-canonical TUFM mitophagy pathway

**DOI:** 10.7150/thno.104124

**Published:** 2025-06-09

**Authors:** Yuling Sun, Wei Wang, Mingyan Li, Wen Guan, Zhimin Gao, Luping Wang, Guanlan Lou, Ao Shen, Jiangbin Wu, Xiyong Yu, Panxia Wang, Xiaoqian Wu

**Affiliations:** 1 Guangzhou Municipal and Guangdong Provincial Key Laboratory of Molecular Target & Clinical Pharmacology, the NMPA and State Key Laboratory of Respiratory Disease, School of Pharmaceutical Sciences, Guangzhou Medical University, Guangzhou 511436, China.; 2 Department of Thoracic Surgery, The Second Affiliated Hospital, Guangzhou Medical University, Guangzhou 510260, China.; 3 Department of Cardiology, Guangzhou Institute of Cardiovascular Disease, Guangdong Key Laboratory of Vascular Diseases, The Second Affiliated Hospital, Guangzhou Medical University, Guangzhou 510260, China.; 4 Institute for Cardiovascular Science and Department of Cardiovascular Surgery of First Affiliated Hospital, Suzhou Medical College, Soochow University, Suzhou, Jiangsu 215031, China.

**Keywords:** mitophagy, RAB7, TUFM, myocardial infarction

## Abstract

**Rationale:** Cardiomyocyte apoptosis critically contributes to ischemic heart failure (IHF) progression. While the endosome-lysosome system governs cellular homeostasis, the functional significance of its master regulator RAB7 in cardiac pathophysiology remains unexplored.

**Methods:** Using myocardial infarction (MI) models via left anterior descending coronary artery ligation in cardiomyocyte-specific RAB7 knockout mice and adeno-associated virus-mediated RAB7 overexpression models, we assessed cardiac function and adverse remodeling through echocardiography and pathophysiological assessment. Mitophagy flux was quantified using mt-Keima mice and confocal imaging. Molecular mechanisms were dissected through immunoprecipitation coupled with mass spectrometry (IP-MS) analysis and molecular experiment validation.

**Results:** RAB7 expression decreased in ischemic myocardium. Cardiomyocyte-specific RAB7 ablation exacerbated while RAB7 overexpression attenuated post-MI cardiac dysfunction and maladaptive remodeling. RAB7 enhanced mitophagic clearance of damaged mitochondria, reducing cardiomyocyte apoptosis under ischemic stress both *in vitro* and *in vivo*. Mechanistically, TUFM, a mitochondrial translation elongation factor, was identified as a novel effector of RAB7. RAB7 facilitated the recruitment of TUFM and LC3 to damaged mitochondria, thereby enhancing mitophagy. TUFM knockdown significantly diminished the protective effects of RAB7 on mitophagy and cardiomyocyte survival. Finally, administration of ML-098, a chemical RAB7 activator, promoted mitophagy and mitigated IHF progression in mice.

**Conclusion:** We identify RAB7 as a novel coordinator of cardioprotective mitophagy through TUFM-mediated machinery assembly. The RAB7-TUFM axis represents a therapeutic target for IHF that warrants further clinical evaluation.

## Introduction

Over the past decades, advancements in timely intervention and treatment have significantly reduced acute myocardial infarction (AMI)-related mortality from over 20% to 12.4% 1. Consequently, the number of AMI survivors is increasing and most of them eventually develop ischemic heart failure (IHF), which boosts the overall HF population globally 2. Although experimental and clinical efforts have yielded therapies that alleviate HF when administered peri-ischemia 3, 4, the molecular mechanisms driving the transition from adaptive to maladaptive cardiac remodeling post-MI remain poorly understood, hindering the development of strategies to prevent HF progression.

Mitochondria, the primary ATP-producing organelles in cardiomyocytes, are critical for maintaining cardiomyocyte redox balance, calcium homeostasis, and survival 5, 6. Mitochondrial dysfunction disrupts these processes, leading to energy depletion, oxidative stress, and ultimately cardiomyocyte death—a hallmark of IHF pathogenesis 6, 7. To sustain mitochondrial integrity, cells rely on dynamic quality control mechanisms, including mitophagy—the selective autophagic clearance of damaged mitochondria. As such, mitochondrial quality control is particularly indispensable for highly metabolic and terminally differentiated cells like cardiomyocytes due to the limited capacity in cell division-dependent dilution of damaged mitochondria 8. Previously, we found mitophagy is inadequate in a mouse model of obesity cardiomyopathy, which contributes to mitochondrial dysfunction and cardiomyopathy 9. Insufficient mitophagy has been implicated in mitochondrial dysfunction across cardiovascular diseases 9, 10, yet strategies to effectively enhance mitochondrial clearance in ischemic cardiomyopathy remain limited.

RAB7, a small GTPase of the Ras-like superfamily, regulates vesicular trafficking within the endosome-lysosome system by cycling between active state (GTP-bound) and inactive state (GDP-bound) 11, 12. Beyond its canonical role in vesicle transport, RAB7 facilitates mitochondria-lysosome interactions in its active form, modulating processes such as apoptosis, autophagy, and mitophagy 13-15. Notably, RAB7 mutations underlie Charcot-Marie-Tooth disease type 2B (CMT2B), a hereditary neuropathy characterized by muscle atrophy and impaired mitochondrial dynamics 16, 17. Despite that RAB7 is assumed to be critically involved in multiple cardiovascular diseases, the specific role of RAB7 in the development and progression of heart diseases remains unclear, such as IHF 15, 18. Inspiringly, a recent study demonstrated that RAB7 deficiency impairs pulmonary artery endothelial function and promotes pulmonary hypertension 19. In addition, TBC1D15, as a RAB7 GTPase-activating protein, has been shown to regulate mitochondrial-lysosome contacts and confer myocardial protection against MI 15, suggesting that RAB7 may play a vital role in the pathophysiology of IHF. However, the exact molecular mechanisms and the role of RAB7 in IHF remain to be elucidated.

In the current study, using a permanent ligation of the left anterior descending (LAD) coronary artery mice model, we demonstrated, for the first time to our knowledge, that cardiomyocyte-specific *Rab7* deletion exacerbated cardiac dysfunction and pathological remodeling, accompanied by impaired mitophagy, mitochondrial dysfunction, and increased cardiomyocyte death. Mechanistically, TUFM, a mitochondrial translation elongation factor, was identified as a novel effector of RAB7. RAB7 facilitated the recruitment of TUFM and LC3 to damaged mitochondria, thereby enhancing mitophagy. Furthermore, both genetic and pharmacological activation of RAB7 attenuated IHF progression, underscoring its therapeutic potential, thus warranting RAB7 as a promising therapeutic target for treating IHF.

## Results

### RAB7 is downregulated in cardiac tissues and cardiomyocytes under ischemic stress

**Figure 1:**
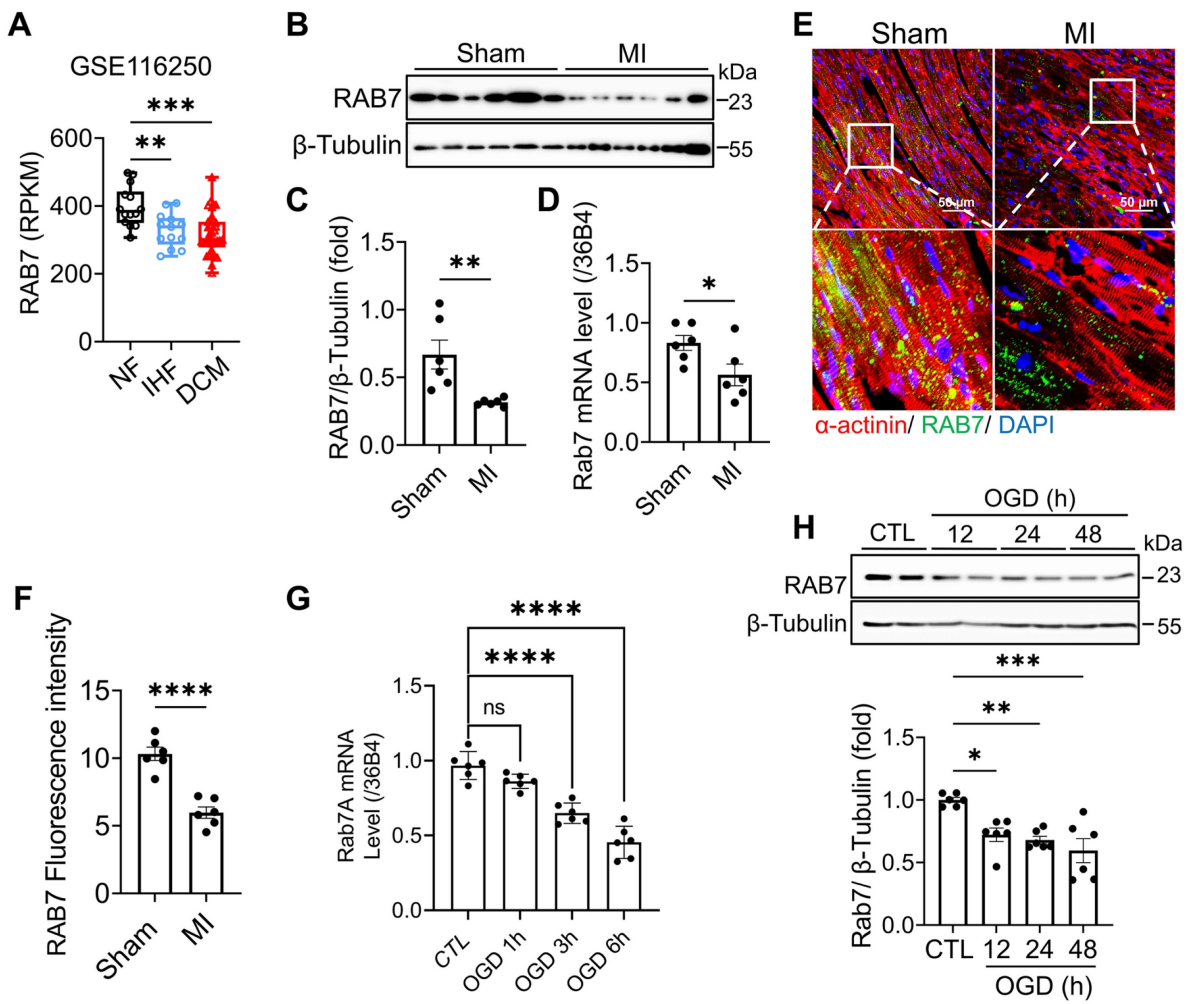
** RAB7 is downregulated in the heart tissues under myocardial infarction and ischemic cardiomyocytes. (A)** Data mining of the publicly available Gene Expression Omnibus database (GSE116250) with the heart tissue of 14 non-failing patients (NF), 13 ischemic heart failure patients (ICM) and 37 dilated cardiomyopathy patients (DCM). **(B)** and **(C)** Representative immunoblots and quantitative analysis of RAB7 protein levels in MI mouse hearts (n = 6). **(D)** Relative mRNA levels of RAB7 in heart tissues from Sham group and MI group (n = 6). **(E)** and **(F)** Representative images of immunofluorescence staining and quantification of RAB7 in peri-infarct area in sham or MI mice are shown (n = 6). Scale bar = 50 μm. ^****^P < 0.0001. **(G)** RAB7 mRNA in OGD-treated NRCMs (n = 3). **(H)** The protein levels of RAB7 in OGD-treated NRCMs (n = 4). All data represent the mean ± SEM. Statistical analysis was evaluated by unpaired Student's t-test **(A-F)** and one-way ANOVA analysis followed by Tukey's multiple comparison test **(G and H)**.^ *^P < 0.05, ^**^P < 0.01, ^***^P < 0.001, ^****^P < 0.0001.

Analysis of the Human Protein Atlas database revealed high RAB7 expression in cardiomyocytes compared to other cell types (Figure S1A). To assess its relevance in human disease, we analyzed cardiac tissues from IHF and dilated cardiomyopathy (DCM) patients in the GEO dataset (GSE116250). RAB7 expression was significantly reduced in IHF and DCM samples compared with non-failing donors (Figure 1A). Furthermore, in a murine myocardial infarction model induced by permanent LAD ligation, RAB7 mRNA and protein expression were significantly decreased post-MI surgery (Figure 1B-D). *In situ* Immunofluorescence staining confirmed that RAB7 is decreased in the cardiomyocytes in the border zone of myocardial infarction surgery (Figure 1E-F).

To validate these findings *in vitro*, neonatal rat cardiomyocytes (NRCMs) were subjected to oxygen-glucose deprivation (OGD) to mimic ischemic injury. Both mRNA and protein levels of RAB7 were downregulated following OGD treatment (Figure 1G-H). Collectively, these data demonstrate that RAB7 expression is suppressed in cardiac tissues and cardiomyocytes under ischemic conditions, implicating its potential role in IHF pathogenesis.

### Cardiac-specific RAB7 deletion accelerated IHF progression under MI

To determine the role of RAB7 during cardiac pathogenesis, we generated cardiomyocyte-specific RAB7-deficient mice (Myh6-Cre; RAB7^fl/fl^, also referred to as RAB7 cKO mice) by crossing *RAB7^fl/fl^* mice with *Myh6-Cre* mice (Figure S2A), confirmed by genotyping PCR (Figure S2B). Immunoblotting assay, qPCR and immunofluorescence staining analyses validated RAB7 depletion specifically in cardiomyocytes but not in other tissues (Figure S2C-E). At baseline, these mice grew normally and did not exhibit any discernable abnormalities for the first 3 months of age (Table S1).

**Figure 2:**
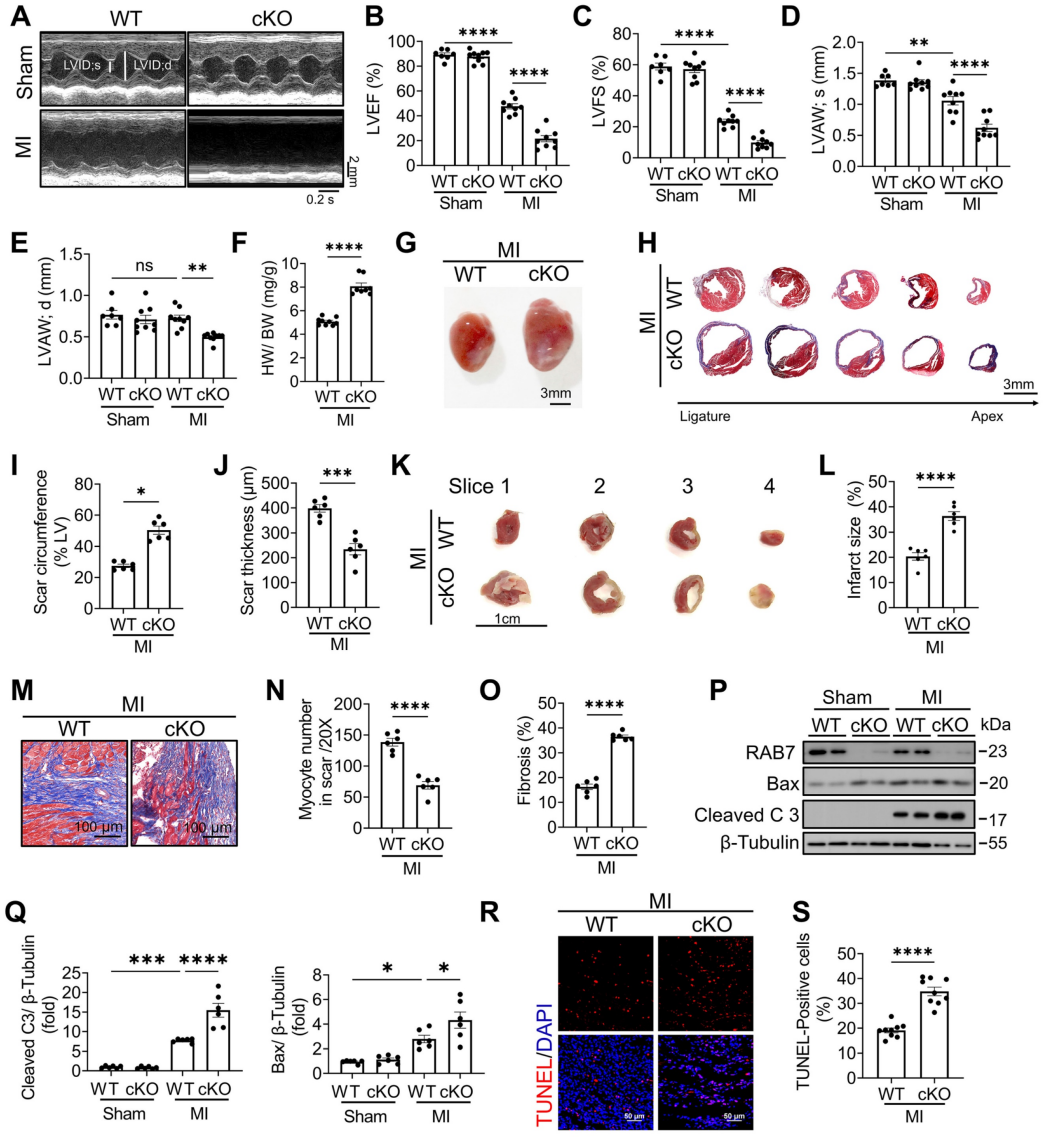
** RAB7 deletion aggravates MI-induced cardiac dysfunction and cardiac remodeling. (A)** Representative left ventricular M-mode echocardiographic tracings in short-axis view in the cardiomyocyte specific *Rab7* deficient mice and the wild type (WT) controls post MI or sham surgery for 14 days. **(B)** Percentage of left ventricular ejection fraction (LVEF), n = 9. **(C)** Percentage of left ventricular fractional shortening (LVFS), n = 9. **(D)** left ventricular end-systolic anterior wall thickness (LVAW; s), n = 9. E, left ventricular end-diastolic anterior wall thickness (LVAW; d), n = 9. **(F)** Heart weight to body weight ratio (n = 6). **(G)** Representative gross images. Scale bar, 3 mm. **(H)** Masson trichrome staining of sequential heart cross sections from each block were cut at 200 μm intervals in indicated groups on day 14 after MI. Scale bar, 3 mm. **(I)** and **(J)** Summarized data of infarct scar circumference and scar thickness at papillary muscle level were measured from sequential sections in H (n = 6). **(K)** 2,3,5-triphenyltetrazolium chloride (TTC) staining. Scale bar, 1 cm. **(L)** Quantitation of infarct size by assessing the TTC -positive areas (n = 6). **(M)** Representative images and **(N-O)**, quantification of Masson's trichrome to assess preserved myocardium and cardiac fibrosis in the infarct border zone on 14 days post MI (n = 6). Scale bar, 100 μm. **(P)** Representative image of Immunoblotting assay of Cleaved caspase 3 and Bax in the heart tissues of the indicated groups on day 5 after MI. **(Q)** Quantification of proteins level in P (n = 6). **(R)** TUNEL staining of the apoptotic cells in the heart tissues in the indicated groups on day 5 after MI. Scale bar, 50 μm. **(S)** Quantification of apoptosis in R (n = 9). Data are shown as mean ± SEM. Statistical analysis was evaluated by unpaired Student's t-test **(F, I, J, L, N, O and S)** and Two-way ANOVA analysis followed by Tukey's multiple comparison test **(B, C, D, E and Q)**. ^*^P < 0.05, ^**^P < 0.01, ^***^P < 0.001, ^****^P < 0.0001.

To explore the role of RAB7 deficiency in the pathological condition, LAD ligation was performed in RAB7 cKO mice and their wild-type littermates. Echocardiographic assay revealed that cardiomyocyte RAB7 deletion exacerbated cardiac dysfunction in the mice under MI as indicated by further reduced left ventricular ejection fraction (LVEF) and fractional shortening (LVFS) (Figure 2A-C), alongside decreased anterior wall thickness (LVAW;s and LVAW;d) (Figure 2D-E), and elevated ventricular diameters (Figure S3 & Table S2). Morphological analysis increased heart weight-to-body weight ratios (HW/BW) and infarct sizes in RAB7 cKO mice at day 14 post-MI (Figure 2F-G). Masson's trichrome staining of serial heart cross sections from apex to the area above the ligation site further demonstrated expanded fibrotic scar areas and reduced viable myocardium in the infarct border zone in the RAB7 cKO mice post MI (Figure 2H-J). 2,3,5-triphenyl tetrazolium chloride (TTC) staining confirmed a larger infarct size in the RAB7 cKO mice (Figure 2K-L & Figure S4). Irreversible death of cardiomyocytes is the main cause of myocardial injury and remodeling 20. Our results demonstrated that RAB7 deletion exacerbated cardiomyocyte loss and cardiac fibrosis in the infarct area post-LAD ligation (Figure 2M-O). Consistent with aggravated remodeling, RAB7 deletion significantly elevated cardiomyocyte death post-MI, as revealed by the increased protein level of cleaved caspase 3 and TUNEL-positive cells (Figure 2P-S).

### Increased expression of RAB7 attenuated pathological LV remodeling post-MI

**Figure 3:**
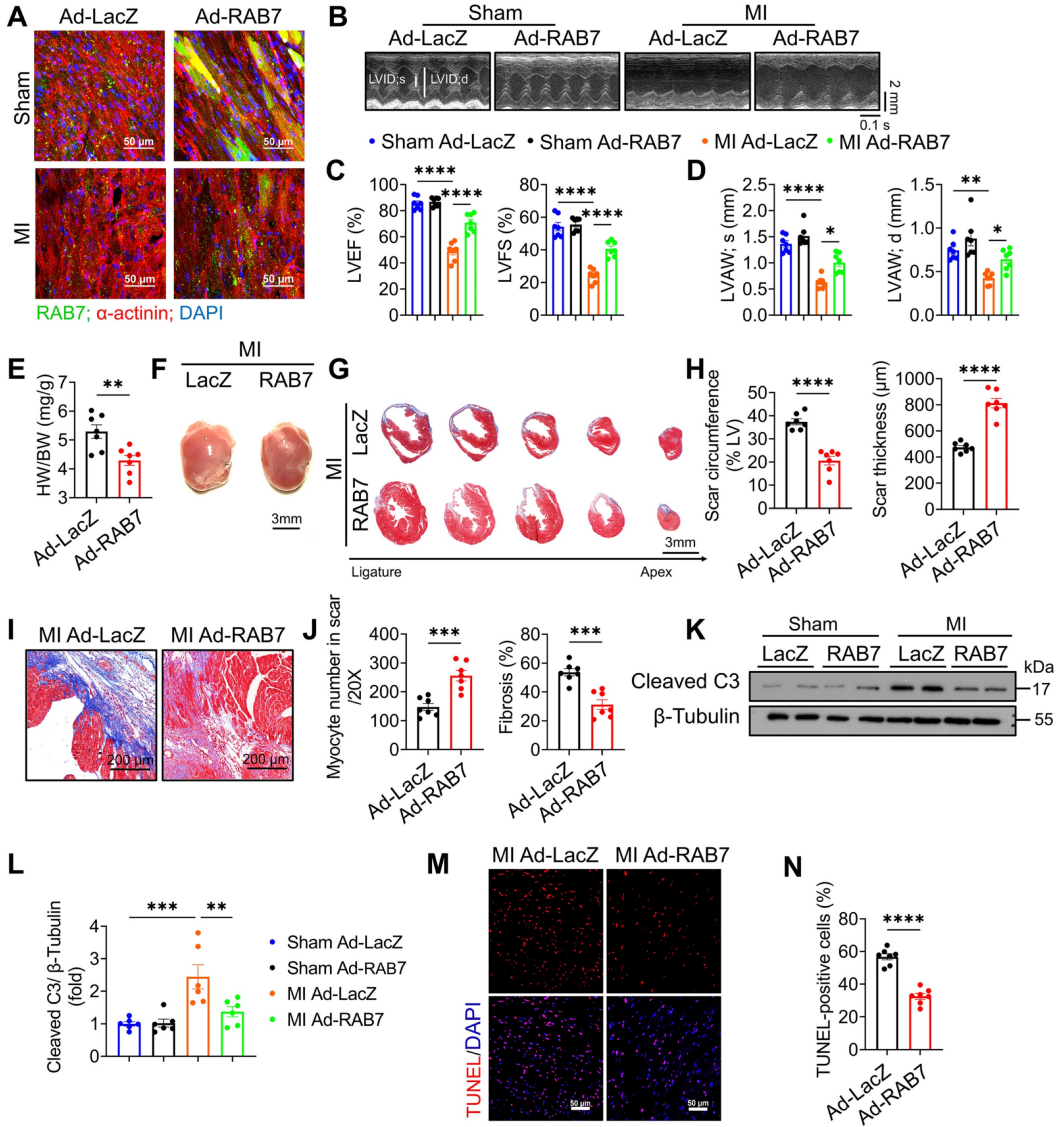
** RAB7 Overexpression protects against cardiac dysfunction and adverse remodeling in IHF.** Adenovirus encoding RAB7 or Ad-LacZ was delivered to left ventricular myocardium via *in situ* multi-point injection post MI and cardiac function and adverse remodeling were assayed. **(A)** Representative images of immunofluorescence staining to indicate successfully overexpression of RAB7 in myocardium. **(B)** Representative M-mode echocardiographic images in indicated groups on day 14 post-MI. **(C)** The results of LVEF and LVFS (n = 7). **(D)** The results of LVAW;s and LVAW;d (n = 7). **(E)** Heart weight to body weight ratio (n = 7). **(F)** Representative gross images of hearts. Scale bar, 3 mm. **(G)** Masson trichrome staining of sequential heart cross sections from each block were cut at 200 μm intervals in indicated groups on day 14 after MI. Scale bar, 3 mm. **(H)** Quantification of infarct scar circumference and scar thickness at papillary muscle level was measured from sequential sections in G (n = 7). **(I-J)** Representative images and quantification of Masson's trichrome to assess the number of myocardium and cardiac fibrosis in the infarct border zone in the mice of indicated groups on day 14 post MI (n = 7). Scale bar, 200 μm. **(K)** Representative image of Immunoblotting assay of Cleaved caspase 3 in the heart tissues of the indicated groups on day 5 after MI. L, Quantification of proteins level in K (n = 4). **(M)** TUNEL staining of the apoptotic cells in the heart tissues in the indicated groups on day 5 after MI. Scale bar, 50 μm. **(N)** Quantification of apoptosis in M (n = 7). Data are shown as mean ± SEM. Statistical analysis was evaluated by unpaired Student's t-test **(F, H, J and N)** and Two-way ANOVA analysis followed Tukey's multiple comparison test **(C, D and L)**. ^*^P < 0.05, ^**^P < 0.01, ^***^P < 0.001, ^****^P < 0.0001.

To further delineate the cardioprotective role of RAB7, adenoviral RAB7 overexpression (*Ad-RAB7*) was achieved via *in situ* myocardial injection post-LAD ligation (Figure S5A). Robust RAB7 expression in the left ventricle was confirmed up to 14 days post-MI (Figure 3A & Figure S5B-C). Echocardiography demonstrated that RAB7 overexpression conferred protection against MI as indicated by the significantly elevated LVEF and LVFS (Figure 3B-C), reduced ventricular dilation, and increased anterior wall thickness compared to *Ad-LacZ*-treated controls (Figure 3D, S6, and Table S3). Furthermore, Ad-RAB7-treated mice exhibited reduced heart weight and HW/BW ratio (Figure 3E-F). Histological assessment revealed preserved myocardial integrity in the infarct border zone, with reduced fibrotic scar circumference and enhanced scar wall thickness (Figure 3G-J). Consistently, RAB7 overexpression mitigated cardiomyocyte apoptosis, as indicated by diminished cleaved caspase-3 levels (Figure 3K-L), and fewer TUNEL-positive cells (Figure 3M-N).

*In vitro*, RAB7 overexpression in the NRCMs augmented cardiomyocyte viability, and reduced cardiomyocyte apoptosis under OGD stress (Figure S7A-C). Conversely, RAB7 silencing exacerbated OGD-induced cytotoxicity, further validating its protective role in cardiomyocyte survival (Figure S8A-C). These data collectively establish that RAB7 overexpression attenuates ischemic injury by preserving cardiac structure and suppressing cell death.

### RAB7 deficiency exacerbates mitochondrial dysfunction post-MI

**Figure 4:**
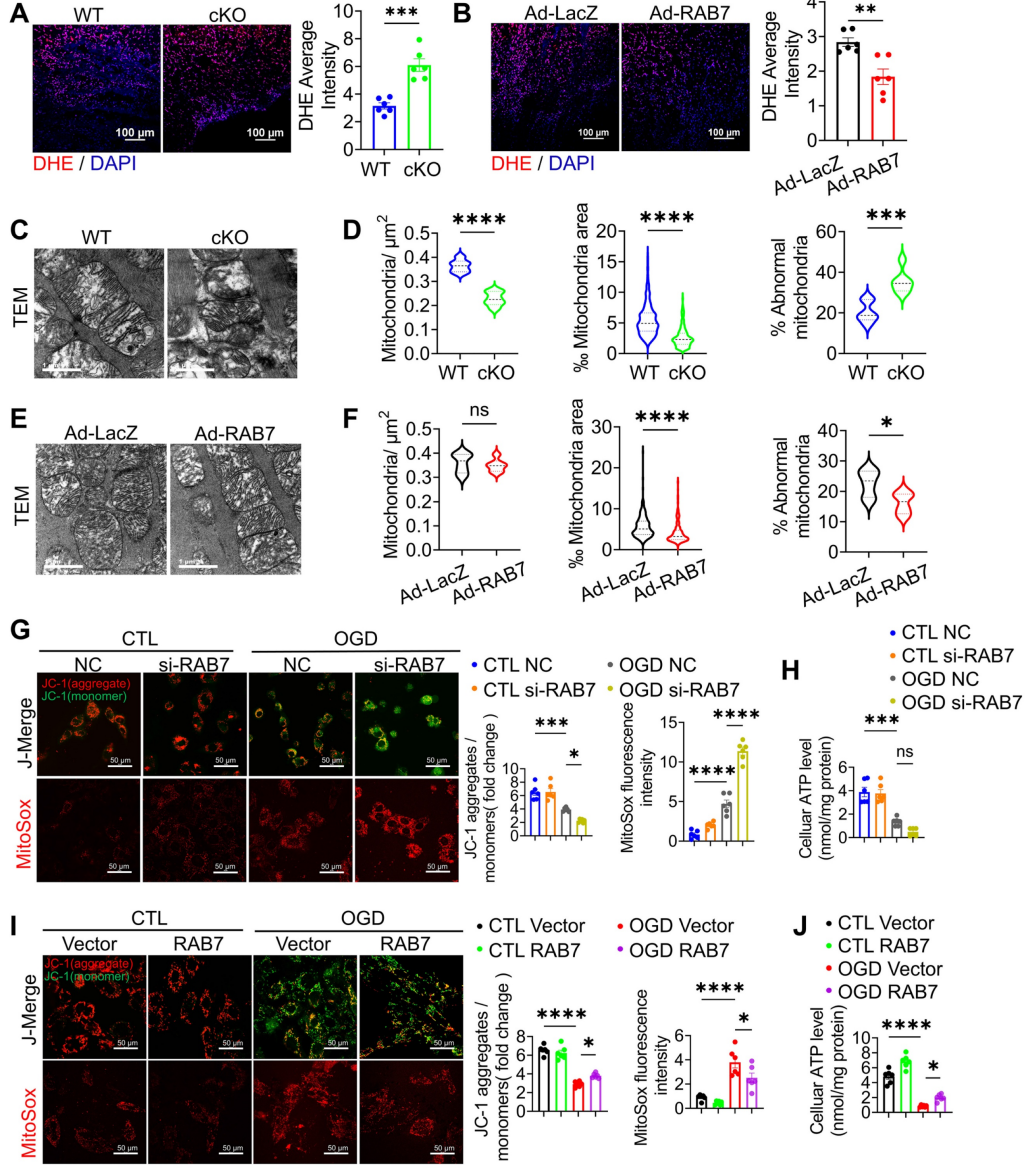
** Cardiomyocyte RAB7 deficiency promoted mitochondrial damage post-MI. (A-B)** Representative image and quantification of DHE staining of the cardiac tissue in the mice of indicated groups on day 5 post-MI (n = 6). Scale bar, 100 μm. **(C)** Representative image of transmission electron microscopy (TEM) observation of the cardiac tissues in the mice of indicated groups on day 5 post-MI. Scale bar, 1 μm.** (D)** Quantification of mitochondrion contents and abnormality based in C. **(E)** Representative TEM image in the mice of indicated groups on day 5 post-MI. Scale bar, 1 μm. **(F)** Quantification based in E. Mitochondria/μm^2^ refers to the average number of mitochondria; ‰ mitochondrial area refers to the ratio of mitochondrial area to image area; % abnormal mitochondria refers to the percentage of abnormal mitochondria in individual samples. (WT, n = 375; cKO, n = 375; Ad-LacZ, n = 323; Ad-RAB7, n = 324). **(G)** Representative image and quantification of mitochondrial potential measured by JC-1 staining (upper panel) and mitochondrial oxidative stress measured by MitoSOX staining (Lower panel), in the NRCMs with or without RAB7 silencing in the presence or absence of OGD injury for 6 hours (n = 5). Scale bar, 50 μm. **(H)** Cellular ATP level was measured in the NRCMs treated as indicated (n = 3). **(I)** Representative image and quantification of mitochondrial potential (upper panel) and mitochondrial oxidative stress (Lower panel), in the NRCMs with or without RAB7 overexpression in the presence or absence of OGD for 6 hours (n = 5). **(J)** Cellular ATP level was measured in the NRCMs treated as indicated (n = 3). Data are shown as mean ± SEM. Statistical analysis was evaluated by unpaired Student's t-test** (A, B, D and F)** and Two-way ANOVA analysis, followed by Tukey's multiple comparison test **(G-J)**. ^*^P < 0.05, ^**^P < 0.01, ^***^P < 0.001, ^****^P < 0.0001.

Given that mitochondrial dysfunction is essential in mediating cardiomyocyte death and IHF progression post ischemia 21, 22, we investigated the effects of RAB7 on mitochondrial function as early as 5-day post-AMI. RAB7 cKO hearts exhibited elevated reactive oxygen species (ROS) levels, as shown by dihydroethidium (DHE) staining, whereas RAB7 overexpression suppressed ROS accumulation (Figure 4A-B). Transmission electron microscopy (TEM) revealed increased mitochondrial swelling and cristae disruption in the infarct border zone of RAB7 cKO mice (Figure 4C-D), contrasting with preserved mitochondrial ultrastructure in RAB7-overexpressing hearts (Figure 4E-F).

In NRCMs subjected to OGD, RAB7 knockdown exacerbated mitochondrial depolarization, ROS overproduction (Figure 4G), and ATP depletion (Figure 4H). Conversely, RAB7 overexpression attenuated these defects, restoring mitochondrial membrane potential (ΔΨm), reducing oxidative stress, and enhancing ATP synthesis (Figure 4I-J). These findings demonstrate that RAB7 is essential for maintaining mitochondrial integrity and bioenergetics under ischemic stress.

### RAB7 protected cardiomyocytes against ischemia via a TUFM-dependent mitophagy pathway

**Figure 5:**
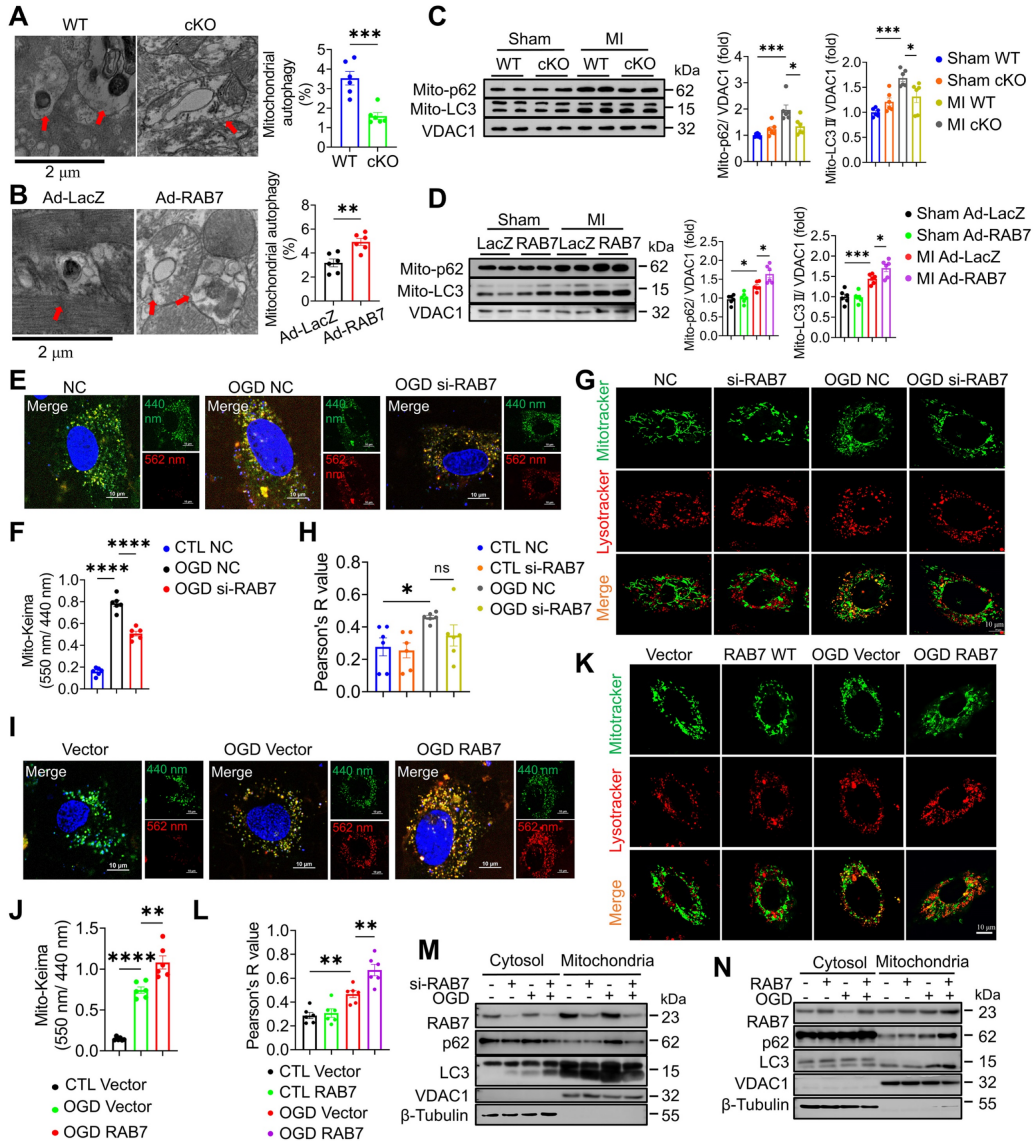
**RAB7 regulated mitophagy post-MI. (A)** Representative transmission electron microscopy (TEM) images and quantification of mitophagosomes in the heart tissue of the RAB7 cKO mice and wild type controls at day 5 after MI (n = 6). Scale bar, 2 μm. Arrows indicate phagosomes containing engulfed mitochondria. **(B)** Representative images and quantitation of immunoblotting assay on LC3 and p62 levels in the mitochondrial fraction of the heart homogenates in the mice with indicated treatment at day 5 after MI (n = 6). **(C)** Representative TEM images and quantification of mitophagosomes in the heart tissue of indicated groups (n = 6). Scale bar, 2 μm. Arrows indicate phagosomes containing engulfed mitochondria. **(D)** Representative images of immunoblotting and quantitation of immunoblotting assay on LC3 and p62 in the mitochondrial fraction of the heart homogenates in the indicated groups (n = 6). **(E)** Mitophagy flux was monitored using Mito-Keima in the NRCMs with or without RAB7 silencing after OGD for 3 hours. Scale bar, 10 μm. **(F)** Quantification of Mito-Keima based in E (n = 5). **(G)** Colocalization of MitoTracker and LysoTracker in the NRCMs with or without RAB7 silencing in the presence or absence of OGD for for 3 hours. Scale bar, 10 μm. **(H)** Quantification of co-localization of Mito-tracker and Lyso-Tracker based in G (n = 5). **(I)** Mitophagy flux was monitored using Mito-Keima in the NRCMs with or without RAB7 overexpression after OGD for 3 hours. Scale bar, 10 μm. **(J)** Quantification of Mito-Keima based in I (n = 5). **(K)** Colocalization of MitoTracker and LysoTracker in the NRCMs of the indicated groups after OGD for 3 hours. Scale bar, 10 μm. **(L)** Quantification of co-localization of Mito-tracker and Lyso-Tracker based in K (n = 5). (M-N) Representative image of Immunoblotting assay of LC3 and p62 in the both cytoplasmic and mitochondrial fractions of the NRCMs with indicated treatment. Data are shown as mean ± SEM. Statistical analysis was evaluated by unpaired Student's t-test **(A, C)** and Two-way ANOVA analysis followed by Tukey's multiple comparison test **(B, D, F, H, J, L)**. ^*^P < 0.05, ^**^P < 0.01, ^***^P < 0.001, ^****^P < 0.0001.

To elucidate the mechanism underlying RAB7-mediated mitochondrial protection, mitophagy was assessed in RAB7 cKO and overexpressing hearts. TEM revealed fewer mitochondria-containing autophagosomes in RAB7 cKO mice post-MI, whereas RAB7 overexpression increased mitophagic vacuoles (Figure 5A-B). Immunoblotting of mitochondrial fractions confirmed reduced LC3-II and p62 levels in RAB7-deficient hearts, indicative of impaired mitophagy (Figure 5C). Conversely, RAB7 overexpression enhanced mitochondrial LC3-II and p62 accumulation (Figure 5D).

Furthermore, mitophagic flux using Mito-Keima fluorescent plasmid and the colocalization of LysoTracker and MitoTracker demonstrated that RAB7 knockdown suppressed OGD-induced mitophagy, while RAB7 overexpression promoted lysosomal degradation of mitochondria (Figure 5E-L). Consistently, the immunoblotting assay demonstrated that RAB7 knockdown significantly reduced OGD-induced LC3II and p62 protein levels in the mitochondrial fraction, while RAB7 overexpression significantly enhanced LC3II and p62 in the mitochondrial fraction (Figure 5M-N; Figure S9).

Moreover, we activated mitophagy using Urolithin A (UA), an mitophagy activator in the RAB7 cKO mice post-MI to evaluate the involvement of mitophagy. UA significantly inducing mitophagy, as shown by increased LC3 and p62 levels in the mitochondrial fraction (Figure S10A-B). Echocardiographic analysis revealed that UA treatment improved cardiac function in RAB7 cKO mice post-MI, as indicated by increased LVEF and LVFS, alongside anterior wall thickness (LVAW;s and LVAW;d) (Figure S10C-F, Table S4). Additionally, UA reduced heart weight and HW/BW ratio, and decreased infarct size, fibrosis and cardiomyocyte death (Figure S10 G-P). These results indicate that UA-induced mitophagy reactivation mitigates cardiac dysfunction and remodeling in RAB7 cKO mice post-MI.

**Figure 6:**
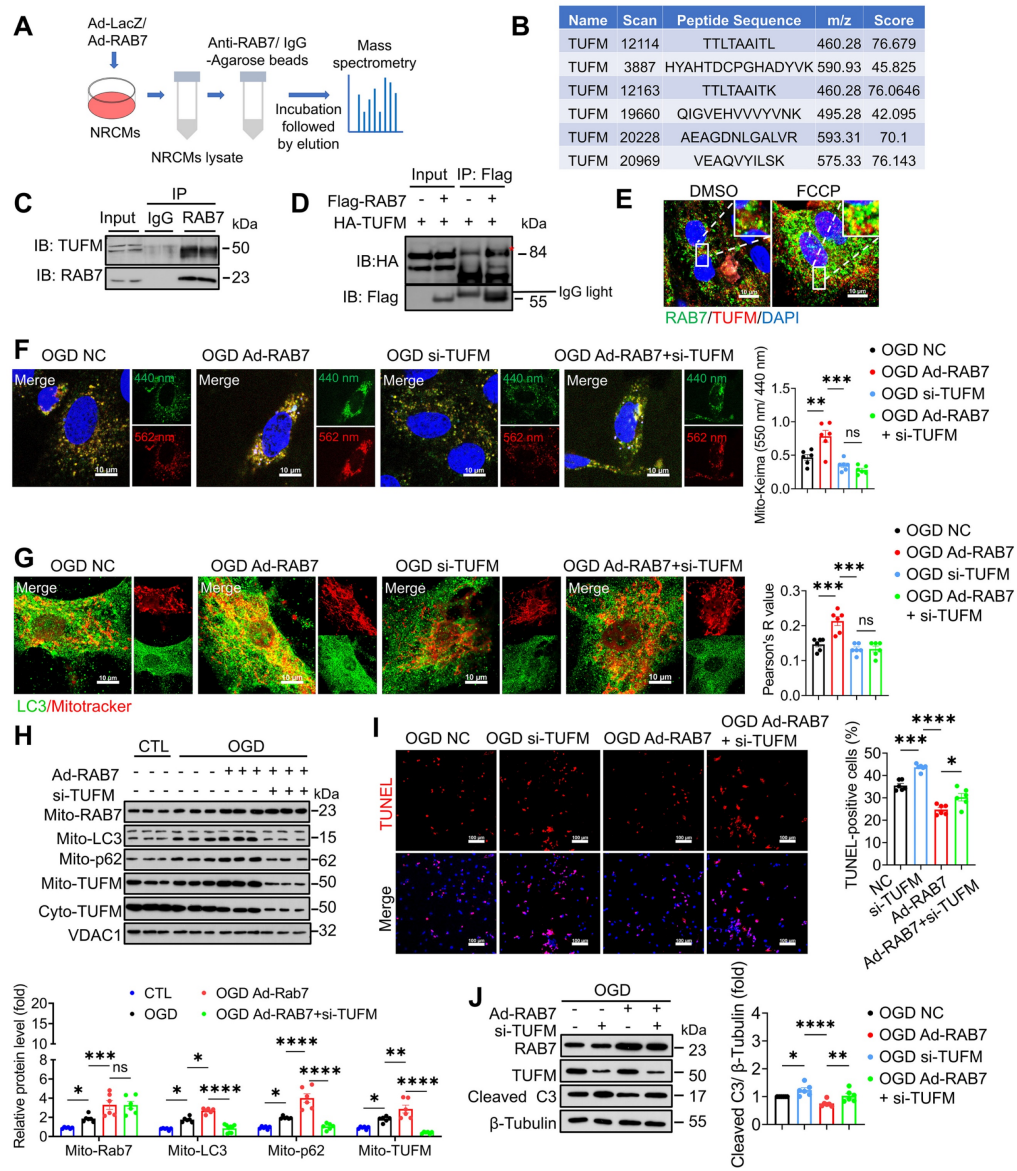
** TUFM mediates the protective effect of RAB7 on mitophagy. (A)** The scheme of immunoprecipitation mass spectrometry. **(B)** The unique peptide fragments of TUFM. **(C)** The representative image of Co-Immunoprecipitation of endogenous RAB7 and TUFM in the NRCMs. **(D)** The representative image of Co-Immunoprecipitation of exogenous RAB7 and TUFM in the HEK239t cells. **(E)** Immunostaining of of RAB7 and TUFM in the NRCMs with or without FCCP (10 μM) treatment. Scale bar, 10 μm. **(F)** The representative image and quantification of Mito-Keima fluorescence in the NRCMs with or without RAB7 overexpression in the presence of TUFM silencing under OGD-stress for 6 hours (n = 6). Scale bar, 10 μm. **(G)** The representative image and quantification of the colocalization endogenous LC3 and mitochondria in the NRCMs (n = 6). Scale bar, 10 μm. **(H)** Representative image of immunoblots and quantitation of LC3, p62 and TUFM in the mitochondrial or cytoplasm fraction of the NRCMs with indicated treatment (n = 6). **(I)** Representative image of TUNEL staining and quantification of the apoptosis in the NRCMs with indicated treatment. Scale bar, 100 μm. **(J)** Representative image of immunoblots and quantitation of of RAB7, TUFM and cleaved caspase 3 in the the NRCMs with or without RAB7 overexpression in the presence of TUFM silencing under OGD-stress for 3 hours. Data are shown as mean ± SEM. Statistical analysis was evaluated by One-way ANOVA analysis followed by Tukey's multiple comparison test.^ *^P < 0.05, ^***^P < 0.001,^****^P < 0.0001.

RAB7 is recruited to the depolarized mitochondria and participates in mitophagy in a Parkin-dependent manner 13. Surprisingly, neither PINK1/Parkin- nor FUNDC1-dependent pathways were altered by RAB7 modulation following OGD stress (Figure S11A-B). We then used immunoprecipitation coupled with mass spectrometry (IP-MS) analysis to identify the protein interactome of RAB7 (Figure 6A), and mitochondrial Tu translation elongation factor (TUFM) was identified as one of the top candidates that interact with RAB7 (Figure 6B). TUFM was a mitochondrion-cytosol dual-localized protein and participated in mitophagy 23, 24. Co-immunoprecipitation (Co-IP) assay was performed and confirmed a direct interaction between RAB7 and TUFM in the NRCMs (Figure 6C) and 293T cells (Figure 6D), which was enhanced by mitophagy induction (FCCP treatment; Figure 6E). TUFM is a nuclear-encoded mitochondrial translation elongation factor playing a critical role in virus-induced mitophagy and participate in host immune response 25, 26. Therefore, we asked whether RAB7 regulated mitophagy via its interaction with TUFM. Notably, Co-IP assay demonstrated that TUFM-LC3 interaction was inhibited under OGD stress, whereas RAB7 overexpression enhanced TUFM-LC3 interaction (Figure S12 A-B). Immunostaining assay confirmed that RAB7 overexpression promote the recruitment of LC3 to the mitochondria, which was inhibited by TUFM knockdown (Figure 6G). Functional studies revealed that TUFM knockdown abolished RAB7-mediated myocardial protection (Figure 6I-J).

Together, our findings supported that RAB7 confers myocardial protection against ischemic stress via a TUFM-dependent mitophagy pathway.

### RAB7 promoted mitophagy via facilitating TUFM recruitment to the damaged mitochondria

**Figure 7:**
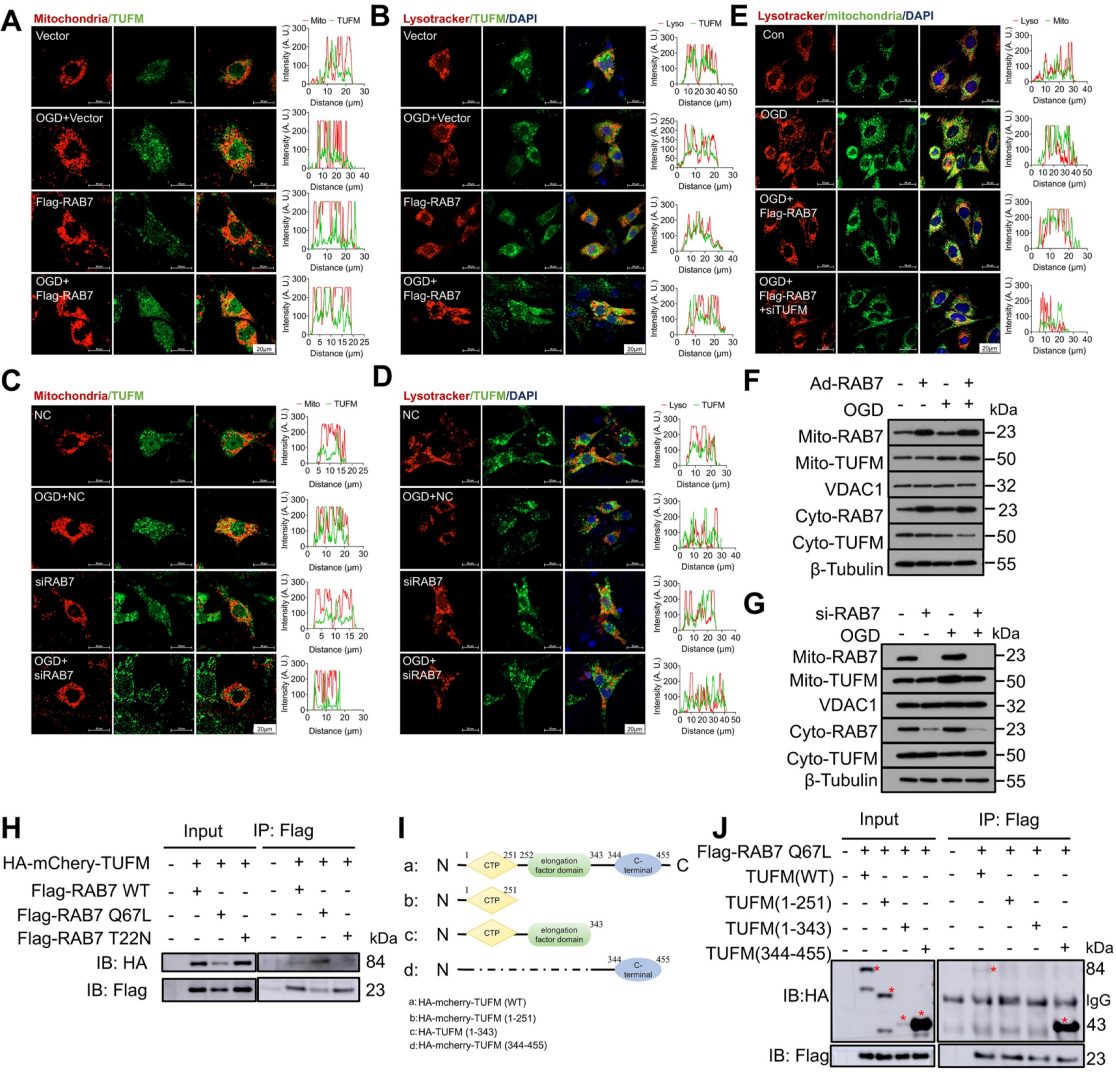
** RAB7 regulates TUFM translocation to damaged mitochondria.** RAB7 was overexpressed or silenced in NRCMs under OGD stress and the colocalization of TUFM with mitochondria or lysosome was measured. **(A-C)**, The colocalization of TUFM with mitochondria. Scar bar, 20 µm. **(B-D)** The colocalization of TUFM with lysosome. Scar bar, 20 µm. **(E)** TUFM was silenced with RAB7 co-overexpression under OGD stress and the co-localization of mitochondria and lysosome was measured. Scar bar, 20 µm. The conditional colocalization analysis for A-E at different spatial distance was carried out using ImageJ software. **(F-G)** The protein expression of TUFM in the mitochondrial fraction of NRCMs was measured. **(H)** The interaction of TUFM with different forms of RAB7. **(I)** Different truncation mutants of TUFM were constructed according to different domain. **(J)** The interactions of RAB7 with these truncation mutants of TUFM were assayed by Co-IP. Red asterisks indicate the position of blots for TUFM mutants.

To dissect the mechanism of RAB7-mediated mitophagy, we first examined whether RAB7 regulates TUFM expression. Total TUFM protein levels were reduced in the infarct border zone post-MI (Figure S13A-D) and in OGD-treated cardiomyocytes (Figure S13E-H), paralleling RAB7 downregulation. However, neither RAB7 deletion nor overexpression altered total TUFM levels *in vivo* or *in vitro*. Considering that TUFM shuttle between mitochondria and cytoplasm 23, 24, and RAB7 is a vesicle transporter, we hypothesized that RAB7 regulates its translocation. In OGD-stressed NRCMs, RAB7 overexpression enhanced TUFM accumulation in mitochondria and lysosomes (Figure 7A-B), whereas RAB7 silencing diminished its organellar localization (Figure 7C-D). Strikingly, RAB7 overexpression increased mitochondria-lysosome contacts under ischemia, an effect abolished by TUFM knockdown (Figure 7E), suggesting TUFM mediates RAB7-driven organelle communication. Mitochondrial fractionation confirmed that RAB7 deficiency blunted OGD-induced TUFM mitochondrial enrichment, while RAB7 over-expression amplified this process (Figure 7F-G, Figure S14A-B). Furthermore, time-course Co-IP revealed progressive dissociation of the RAB7-TUFM complex during prolonged OGD, particularly in the cytosol (Figure S15A-C). Concomitantly, RAB7-TUFM colocalization in mitochondria and lysosomes decreased over time post-OGD (Figure S15D-E), suggesting TUFM recruits RAB7 to damaged mitochondria early in ischemia to initiate clearance.

As RAB7 activity depends on its GTP-bound state 27, we tested whether GTP hydrolysis regulates RAB7-TUFM interaction. We co-transfected wild-type TUFM with three different active forms of RAB7 plasmids (wild-type RAB7 WT, active GTP-bound RAB7 Q67L mutant plasmid, and inactive GDP-bound RAB7 T22N mutant plasmid) into 293T cells. Co-IP assay demonstrated stronger binding between TUFM and the constitutively active RAB7-Q67L mutant compared to the inactive RAB7-T22N mutant or wild-type RAB7 (Figure 7H). Domain mapping identified the C-terminal region of TUFM (344-455 aa) as essential for RAB7 binding (Figure 7I-J). These data establish that GTP-bound RAB7 interacts with TUFM via its C-terminal domain to orchestrate mitochondrial-lysosome crosstalk, enabling mitophagy under ischemic stress.

### Pharmacological activation of RAB7 attenuates IHF progression post-MI

**Figure 8:**
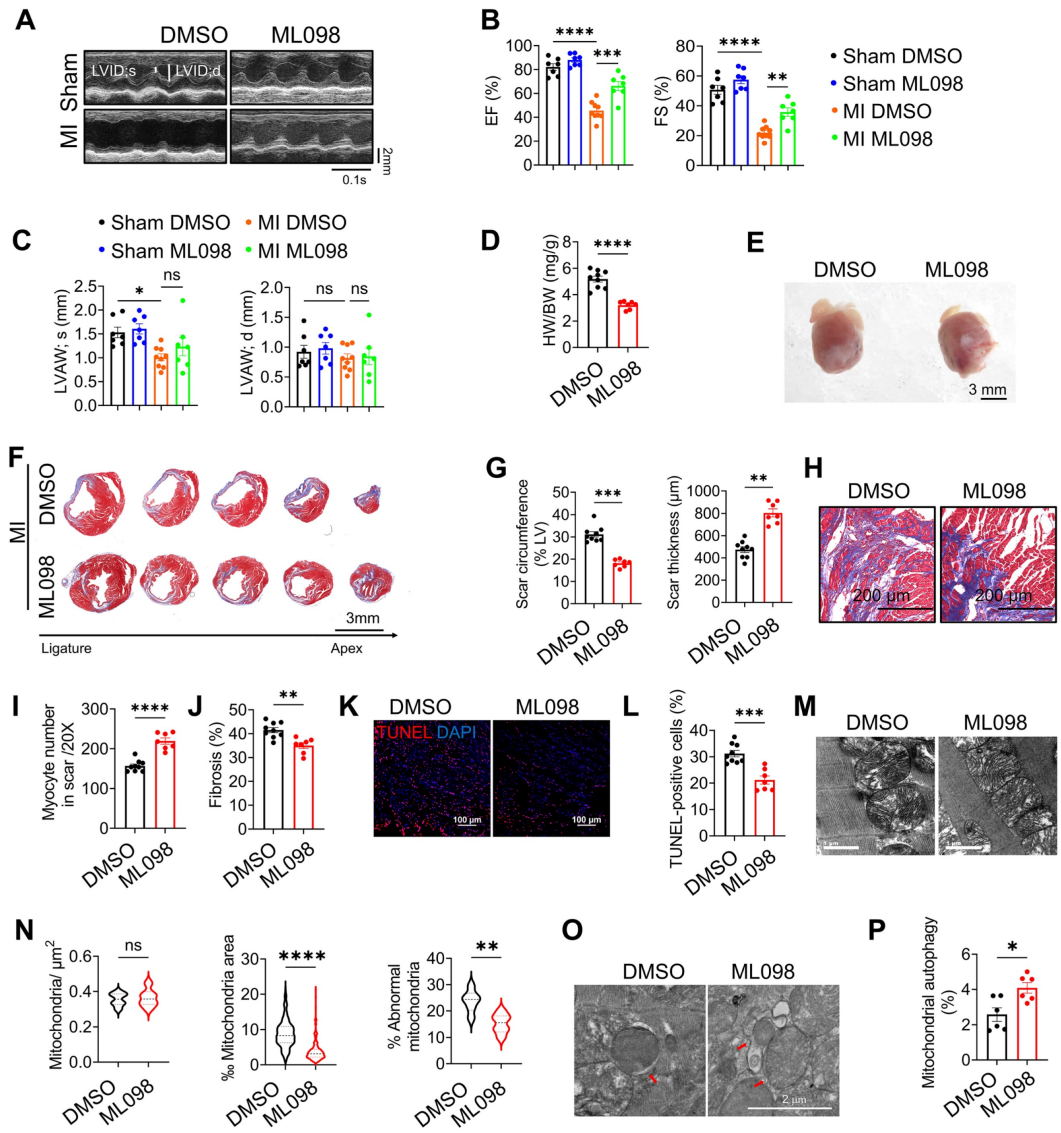
** Pharmacological activation of RAB7 attenuates IHF progression post MI.** ML098 was injected to the mice intraperitoneally post MI for 14 days. **(A)** Representative images of echocardiography. **(B)** The quantification of LVEF, LVFS of the mice from different groups (n = 7). **(C)** LVAW;s and LVAW;d (n = 7). **(D)** The heart weight to body weight ratio in different groups (n = 7). **(E)** Representative images of the gross heart with or without ML098 treatment post MI. Scale bar, 3 mm. **(F)** Masson trichrome staining of sequential heart cross sections from each block were cut at 200 μm intervals in indicated groups on day 14 after MI. Scale bar, 3 mm. **(G)** Quantification of scar circumference and scar thickness at papillary muscle level based in F (n = 7). **(H)** Representative images of Masson's trichrome in the infarct border. Scale bar, 200 μm. **(I)** Quantification of preserved myocardium in the infarct border based in H (n = 7). **(J)** Quantification of cardiac fibrosis based in H (n = 7). **(K)** Representative image of TUNEL staining in the infarct border (n = 7). Scale bar, 100 μm. **(L)** Quantification of cell apoptosis based in K. (M) Representative images of TEM. **(N)** Quantification of mitochondrial contents and abnormality based in M (n = 285; ML098 + MI group, n = 286). Scale bar, 1 μm. **(O)** The representative image of TEM in the heart post MI with or without ML098 treatment. Scale bar, 2 μm. **(P)** Quantification of the engulfed mitochondria by autophagosomes based in O (n = 6). Data are shown as mean ± SEM. Statistical analysis was evaluated by unpaired Student's t-test **(D, G, I, J, L, N and P)** and two-way ANOVA analysis followed by Tukey's multiple comparison test **(B and C)**. ^*^P < 0.05, ^**^P < 0.01, ^***^P < 0.001, ^****^P < 0.0001.

To evaluate the therapeutic potential of RAB7, mice post-MI were treated with ML-098, a small-molecule RAB7 activator 19. ML-098 administration (30 mg/kg/day, intraperitoneal) significantly improved cardiac function, as evidenced by elevated LVEF and LVFS, reduced ventricular dilation, and increased anterior wall thickness compared to vehicle-treated controls (Figure 8A-C, Figure S16 and Table S5). Morphologically, ML-098 reduced HW/BW ratio (Figure 8D-E) while increasing the thickness of the left ventricular anterior wall (Figure 8F-G). Furthermore, ML-098 reduced cardiomyocyte loss and fibrosis in the infarct area post-MI (Figure 8H-I) and significantly alleviated cardiomyocyte death after MI (Figure 8J-K). Moreover, mitochondrial ultrastructural damage, as demonstrated by TEM showing reduced mitochondrial swelling and cristae disorganization (Figure 8L-M). Additionally, ML-098 enhanced mitophagic activity, with increased mitochondria-containing autophagosomes observed in cardiac tissues (Figure 8N-O). These results collectively demonstrate that pharmacological activation of RAB7 ameliorates post-MI cardiac dysfunction by restoring mitochondrial quality control and suppressing maladaptive remodeling.

## Discussion

Comprehending the molecular mechanisms that underlie the transition from adaptive to maladaptive remodeling post-myocardial infarction is crucial for developing efficient strategies for conquering IHF. RAB7 GTPase is a key regulator of membrane trafficking that integrates both membrane trafficking and intracellular signaling 28. Besides its role in the endosome-lysosome system, RAB7 was shown to involve in the autolysosome degradation of specific proteins 14, 15. However, very limited studies evaluate whether RAB7 contributes to pathological cardiac remodeling in IHF progression. In this study, we found RAB7 is significantly decreased in the infarct border and the cardiomyocyte with OGD injury, and cardiomyocyte-specific deletion of RAB7 exacerbated cardiac dysfunction and dysplastic remodeling of the heart post-MI. Meanwhile, we identified TUFM, a mitochondrial translation elongation factor, as a novel binding partner of RAB7 to promote mitophagy. RAB7 conferred protection against myocardial ischemia by facilitating TUFM recruitment to the damaged mitochondria and promoting mitophagy. Importantly, our results indicated that genetic and pharmacological restoration of RAB7 protected against MI-induced heart failure, highlighting RAB7 as a promising therapeutic target for IHF.

RAB7 regulates vehicle trafficking and endocytosis, modulating various cellular biological processes including vesicle-specific transport, organelle interactions, metabolic responses, signal transduction, and autophagy 28, 29. RAB7 mutations in humans cause CMT2B disease, a rare dominantly inherited peripheral neuropathy 30. Beyond axonal neuropathy 31, 32, RAB7 is implicated in pulmonary hypertension 19 and podocytopathy 33. Our study expanded the understanding of the function of RAB7 in ischemic heart failure. Our study expands the understanding of RAB7 function by demonstrating its critical role in ischemic heart failure. We observed reduced RAB7 expression in the infarct border zone of mice, human IHF cardiac tissue, and cardiomyocytes after OGD treatment, suggesting RAB7's involvement in IHF progression. This is the first direct evidence showing reduced RAB7 in failing hearts contributes to maladaptive changes post-MI. Genetic deletion of RAB7 worsened cardiac dysfunction and remodeling post-MI, while overexpression protected against IHF progression. Importantly, RAB7 deletion exacerbated mitochondrial dysfunction, whereas its overexpression maintained mitochondrial homeostasis. Consistent with our findings, disruption of RAB7 function with its mutants impairs mitochondrial function and compromises axonal viability 34. Despite the controversial explanations on CMT2B caused by the missense mutation of RAB7, mitochondrial dysfunction was observed in CMT2B 35, which supports our findings in the cardiomyocyte-specific RAB7 deficient mice. Mingming Sun et al. demonstrated that nuclear dot protein 52 (NDP52) protected against myocardial ischemia through increasing interaction of TBK1 and RAB7 14. TBC domain family member 15 (TBC1D15), as a RAB7 GTPase-activating protein was shown to regulate mitochondria-lysosome contacts and subsequent myocardial protection against ischemia 15. Although the specific effect of RAB7 in cardiomyocytes is not yet fully understood in these studies, they additionally support our finding of the crucial role of RAB7 against IHF progression. Importantly, we integrated both genetic and pharmacological activation strategies, showing that restoring the expression or the activity of RAB7 following MI is resistant to cardiac dysfunction, pathological remodeling, and HF progression.

Mitochondrial quality control, especially mitophagy, were closely related to the progression of cardiovascular diseases 36, 37. There are two main mechanisms of mitophagy: PINK1-Parkin-mediated ubiquitin-mediated mitophagy and the FUN14 domain-containing protein 1 (FUNDC1) receptor-mediated pathway. A previous study showed that active GTP-bound RAB7 promoted mitochondria-lysosome contact in HeLa cells and regulated mitochondria fission instead of mitophagy 27. However, the role and the detailed mechanism of RAB7 on mitophagy in the cardiomyocytes are still unclear. In this study, we found that cardiomyocyte-specific deletion of RAB7 exacerbated cardiac dysfunction post-MI, concurrently with impaired mitophagy. RAB7 promoted the clearance of damaged mitochondria post-MI and maintained mitochondrial hemostasis. Strikingly, UA-induced mitophagy activation can mitigate cardiac dysfunction and pathological remodeling in RAB7-deficient mice following myocardial infarction, confirming the involvement of mitophagy in the protective role of RAB7 against IHF. However, these beneficial effects depend on neither Parkin-mediated nor receptor-mediated pathways.

RAB7 functions as a molecular switch in the endosome-lysosome system and signaling transduction through GTP/GDP transition, by interacting with various effectors, such as motors, kinases, and adaptors 38. In this study, by combining Liquid chromatography-tandem mass spectrometry and coimmunoprecipitation assay, TUFM was identified as a novel effector of RAB7 to promote mitophagy in the cardiomyocytes. As a mitochondria-cytosol dual-localized protein, TUFM is an autophagy effector and is essential for mitochondrial function by regulating mitochondria clearance in human cells 26, 39. Here, we found that active GTP-bound RAB7 interacts at the C-terminal of TUFM and the co-localization of RAB7 with TUFM increased following mitophagy activation. Silencing TUFM inhibited the mitophagy enhancement by RAB7 overexpression under OGD stress and consequently hindered the protective effects of RAB7 on mitochondrial homeostasis and cardiomyocyte death. RAB7 knockdown disrupted the localization of TUFM to mitochondria and lysosomes under ischemic stress, while RAB7 overexpression significantly increased the localization of TUFM to both organelles. Similarly, a recent study supports that TUFM could translocate from cytoplasm to mitochondria and promote mitophagy 23, 24. Our results demonstrated that the interaction between TUFM and LC3 II was decreased in cardiomyocytes subjected to OGD. However, RAB7 overexpression increased this interaction, thereby promoting mitophagy. This finding provides direct evidence that TUFM senses damaged mitochondria in cardiomyocytes and acts as an effector of RAB7 to facilitate the engulfment of damaged mitochondria by autophagosomes for lysosomal clearance. Importantly, this TUFM-mediated mitophagy pathway is distinct from the canonical Parkin-dependent or receptor-mediated pathways, as RAB7 does not alter these conventional mitophagy mechanisms. Instead, RAB7 directly interacts with TUFM, enhancing mitophagy as evidenced by increased Mito-Keima and LC3 localization in the mitochondria, effects that were blunted by TUFM silencing. Thus, our study reveals a novel RAB7-TUFM axis that governs mitophagy and mitochondrial quality control in cardiomyocytes, highlighting its potential as a therapeutic target for ischemic heart failure.

## Materials and Methods

All animal experiments and procedures complied with the requirements of the Guide for the Care and Use of Laboratory Animals (NIH Publication No.85-23) and were approved by the Animal Ethics Committee of Guangzhou Medical University. All the mice were housed in a pathogen-free, temperature-controlled environment within a range of 21-23 °C with 12-h light/12-h dark cycles and were given a normal diet and water ad libitum. All animal experiments were performed on age-matched mice, and the mice were randomly assigned to experimental and sham control groups. Investigators followed standard laboratory procedures of randomization and were blinded to the genotypes of the individual animals during the experiments and outcome assessments.

### Generation of transgenic mice with deletion RAB7

The C57BL/6J *RAB7^flox/flox^* mice, which were purchased from the Jackson Laboratory (Stock No. 021589), possess *loxP* sites flanking exon 1 of the *RAB7* gene. The *RAB7^flox/flox^* mice were bred by crossing with Myh6-cre transgenic mice (Jackson Laboratory, Stock No. 011037) to generate cardiomyocyte-specific RAB7-deficient mice, Myh6-cre RAB7*^flox/flox^* mice (RAB7-cKO mice), and their littermates, the RAB7-wild type control mice (WT). All animal care and procedures were approved by the University Committee on Animal Resources at Guangzhou Medical University.

### Statistical analysis

Data are expressed as Mean ± SEM. Statistical analysis was performed using GraphPad Prism 9. The differences between two groups were analyzed using Student's unpaired *t*-test. Multiple groups were compared using One-way ANOVA or Two-way ANOVA with Tukey's multiple comparison test. P values less than 0.05 were considered significant.

All data that support the findings of this study are available in the article and the Supplemental Material. Detailed Materials and Methods are provided in the Supplemental Material.

## Supplementary Material

Supplementary materials and methods, figures and tables.
