# Supplementary material for: RAB7 protects against ischemic heart failure via promoting non-canonical TUFM mitophagy pathway
Source: Theranostics. 2025 Jun 9;15(14):6753–67. doi: 10.7150/thno.104124 (PMC12203679; doi:10.7150/thno.104124)
Supplement: Supplementary file 1 — Supplementary materials and methods, figures and tables. [file thnov15p6753s1.pdf]

## Online Supplementary Material

# RAB7 protects against ischemic heart failure via promoting non-canonical TUFM mitophagy pathway

## Material and Methods

### ***Recombinant adenovirus construction***

Wild-type RAB7-encoding plasmid and the recombinant adenoviral vector harboring human RAB7 cDNA and humanized recombinant red fluorescent protein (mcherry) driven by the cytomegalovirus promoter was prepared by the Genechem (Shanghai, China). A corresponding virus with null content was used as a control.

### ***MI surgery.***

RAB7-cKO mice and their littermate controls (WT) were subjected to left anterior descending artery or sham surgery as we described obviously [1, 2]. Briefly, the mice were anesthetized via continuous inhalation of isoflurane (2 %) and placed on a heated surgical board with all efforts to minimize suffering. In the supine position, the trachea was intubated and the mice were ventilated with a small animal respirator. After pericardiectomy, the left anterior descending coronary artery was tied with an 8-0 silk suture permanently. Sham-treated animals underwent the same surgical procedure, but a suture is passed under the LAD coronary artery without ligation.

To achieve cardiac-specific overexpression of RAB7, adenovirus expressing RAB7 was directly in situ injected into three different sites of the ischemic area at a dosage of $1.0\times10^9$ pfu/μL. Mice transfected with empty adenovirus (Ad-LacZ, control viral vector) were used as a control group.

For pharmacological activation of RAB7 in the mice, ML-098 (HY-19800, MedChemExpress) or the vehicle were injected intraperitoneally at a dosage of 200 μL ML-098 (1μM) daily for 2 weeks immediately after MI. Each mouse was injected with 200 μL every other day. Mice were eventually euthanized at the indicated time points

to obtain heart tissue for histological and biochemical analysis.

***Echocardiography***.

As we have described previously [1, 2], the mice were anesthetized via a continuous inhalation of 1.5% isoflurane and echocardiography was performed using an echocardiographic monitor (Vevo 2100 Mouse Imaging System). M-Mode echocardiography on short axis was used to assess ventricular function. Ultrasound data was measured using the LV Trace tool of the Cardiac Package. Fractional shortening was calculated by the following formula: FS (%) = (LV end diastolic diameter - LV end systolic diameter) / (LV end diastolic diameter) *100 %. The left ventricular end-systolic anterior wall thickness (LVAW; s), left ventricular end-diastolic anterior wall thickness (LVAW; d), left ventricular end-systolic diameters (LVID; s), LV end-diastolic diameters (LVID; d), left ventricular fraction shortening (LVFS), left ventricular ejection fraction (LVEF), cardiac output (CO), left ventricular end-diastolic volume (volume; d) and left ventricular end-systolic volume (volume; s) were measured in 5 consecutive cardiac cycles. Representative M-mode echocardiographic images were chosen based on their quality and to most accurately represent the group average.

***Mouse cardiac tissue collection and processing***

The mice were anesthetized via intraperitoneal injection of pentobarbital sodium for euthanasia before cervical dislocation. Heart tissues were isolated and rinsed with phosphate-buffered saline (PBS), followed by the removal of atrial and vascular connective tissue. Heart weight and body weight are recorded. The heart tissue sections are immediately frozen in liquid nitrogen and then stored at -80 °C. The ventricular tissue was embedded with 4 % paraformaldehyde for further histological analysis.

***TTC staining***

As we described previously [3], the harvested heart of mice was rinsed with PBS, cut into 1 mm thickness sections, and stained with 1% triphenyl tetrazolium chloride

(TTC, Sigma, USA) solution for 15 minutes at 37 °C, followed by 4 % paraformaldehyde fixation for 24 hours. The infarct size (IS, characterized by absence of staining), the area at risk (AAR, characterized by red tissue staining) and non-ischemic area (characterized by blue tissue staining) was analyzed using Image Pro analysis software.

***Quantitative real-time PCR (qPCR)***

Total RNA was extracted from NRCMS using AG RNAex Pro Reagent (AG2110, Accurate Biotechnology) and real-time PCR was performed as we described [3]. To accurately measure gene expression, the primer sequences used for PCR amplification are shown in Online Table I. 36B4 was used as a reference endogenous gene for normalization.

***Immunofluorescence staining***

Frozen tissues were sectioned, hydrated, ruptured, and subsequently blocked with 10 % goat serum for 30 minutes. Then, the tissues were incubated with primary antibodies: α-actinin (dilution 1:100, BM0003, Boster Bio, Wuhan, China) or RAB7 (dilution 1:100, ab137029, Abcam, USA) overnight at 4 °C and then further incubated with fluorescent secondary antibodies (Alexa Fluor 488 or Fluor 594) for 1.5 hours at room temperature (RT). TUNEL staining and DHE staining were performed according to the instructions (Roche, USA). 4', 6-diamidino-2-phenylindole (DAPI, 10 μg/mL, Solabol) stained for 15 min was used to indicate the nucleus.

In vitro, cells were fixed with 4 % paraformaldehyde for 10 min at RT, permeabilized with 1 % Triton X-100 for 20 min, and blocked with 10 % goat serum for 30 min. Cells were incubated with primary antibodies overnight at 4 °C and with secondary antibodies (Alexa Fluor 488 or Fluor 594) for 1.5 hours at RT. DAPI was used to stain the nucleus. Images were captured using a NIKON A1R confocal microscope (Nikon Instruments Inc). The conditional colocalization analysis at different spatial distance was carried out using ImageJ software, which was applicable

to images of both punctate and non-punctate objects.

***Masson's trichrome staining***

The hearts of mice were removed, fixed with 4 % paraformaldehyde for 24 hours at RT, then dehydrated and waxed to prepare paraffin-embedded blocks. 5 μm thick sections were cut with a microtome. Collagen deposition was determined by Masson's trichrome staining. After staining nuclei with Wiegert's iron hematoxylin staining solution (Servicebio) for 5 minutes, the sections were rinsed three times with distilled water and then stained with 0.7 % Masson-Ponceau acid purplish red staining solution for 10 min. After rinsing in 2 % glacial acetic acid, the samples were differentiated in phosphomolybdic acid for 4 min. Sections were then stained with 2 % aniline blue dye solution, incubated sequentially in 100 % ethanol for 5 min and in xylene for 5 min, and then sealed with neutral glue. Images of Masson staining results were finally acquired with a light microscope. Collagen fibers were stained blue, while normal myocardium was stained red.

Referring to previous studies, we used scar circumference and scar thickness expressed as infarct size and left ventricular myocyte/fibrosis ratio expressed as myocyte area to fibrosis area ratio [4].

***Isolated neonatal rat cardiomyocytes （NRCMS） and cell culture***

As we previously described [5], the hearts were removed from 2-day-old Sprague-Daley rat, minced into partials in DMEM medium, and then digested by trypsin overnight at 4 °C and collagenase at 36 °C. DMEM medium containing 15 % fetal bovine serum (FBS) was added to the cell suspension to stop the digestion. This process was repeated several times until all tissue disappeared. The cell suspension was filtered and centrifuged at 1000 rpm for 5 min. The collected cells were resuspended in a DMEM medium containing 15 % FBS. Consequently, cells were incubated at 37 °C in a humidified incubator containing 5 % $CO_2$ for 1 hour. NRCMS suspension was collected and transferred to a new dish. After 24 hours of incubation, NRCMS were

further induced by OGD stimulation.

Human embryonic kidney (HEK) 293T cells were cultured in DMEM medium containing 10 % FBS, 100 U/mL penicillin, and 100 mg/mL streptomycin and incubated in a humidified incubator containing 5 % $CO_2$.

***RNA Interference***

NRCMS were transfected with negative control (NC), siRNA targeting RAB7, or siRNA targeting TUFM. siRNA molecules were synthesized by RiboBio (Guangzhou, China). The siRNA sequence targeted RAB7 was 5'- CCAGTACAAAGCCACAATA - 3'. The siRNA sequence targeted TUFM was 5'- CGACAAGCCACATGTGAAT - 3'. Different siRNA (50 nM) was transfected into NRCMS for 24 h or 48 h to induce the silencing of targeted protein by using Lipofectamine RNAiMAX (Invitrogen, USA).

***Recombined plasmids construction and transfection***

All plasmids used in this study were constructed or purchased from Weizhen Biosciences Inc (Shandong, China) including wild-type flag tag RAB7 plasmid (WT-RAB7), GFP-Flag-RAB7 Q67L mutant plasmid, GFP-Flag-RAB7 T22N mutant plasmid, mKeima-Red-Mito adenovirus, mcherry-HA-TUFM (WT) (84.48 KDa), mcherry-HA-TUFM(1-251 aa) (60 KDa) truncation mutant plasmid, HA-TUFM(1-343 aa) (45 KDa) truncation mutant plasmid and mcherry-HA-TUFM(344-455) (43.44 KDa) truncation mutant plasmid. For 6 well plates, plasmids/well (2 μg) were transfected into cells by using PolyJet transfection reagent according to the instruction for 24 h or 36 h

***Oxygen and glucose deprivation (OGD)***

OGD was performed as we described previously [3]. Briefly, the cultured medium of NRCMS was replaced by serum-free, glucose-free F-12 (Gibco) medium and cultured in an anoxia chamber (InVivo 500, Ruskinn Life Science) saturated with $94\%N_2/5\%CO_2/1\%O_2$ at 37 °C for the indicated time.

***JC-1 and MitoSox staining***

The mitochondrial potential was monitored by 5,5′,6,6′-Tetrachloro-1,1′,3,3′-tetraethyl-imidacarbocyanine iodide staining (JC-1, Beyotime, Shanghai, China) according to the instruction. To monitor the reactive oxygen species (ROS) in mitochondria, NRCMS were incubated with MitoSOX (5 μM, M36005, Invitrogen, USA) at 37 °C for 10 min and washed twice with PBS. The fluorescence was observed by using a NIKON A1R confocal microscope and quantified by using Image Pro software.

***Mitophagic flux analysis***

Mito-Keima plasmid was purchased from Weizhen Biosciences Inc. (Shandong, China). As previously described, Mito-Keima is used to assess mitophagic flux [6]. It is a mitochondria-targeted, pH-sensitive, dual-excitation ratiometric fluorescent protein that is resistant to lysosomal degradation. The shorter-wavelength (440 nm, green) excitation predominates in the mitochondria environment (pH 8.0), whereas the longer-wavelength (578 nm, red) excitation predominates in the acidic lysosome environment (pH 4.5). The NRCMS were transfected with Mito-Keima plasmid for 16 h, then the medium was changed to suffer from OGD stress for 24 h. DAPI was used to indicate the nucleus of NRCMS. Fluorescences at different wavelengths (405, 488 nm, and 562 nm) were observed using a confocal microscopy, and quantified by using Image Pro software.

***Mitochondria fractionations***

Mitochondrial proteins from heart tissue were prepared using Cell Mitochondria Isolation Kit (C3606, Beyotime, China). 100mg fresh tissue was minced and washed with PBS. Add trypsin-EDTA Solution and ice for 20 min. Centrifuge at 600 g for 10 s, discard supernatant, and add mitochondrial isolation reagent A. Centrifuge at 600 g for 10 s and discard supernatant. Mitochondrial isolation reagent A containing PMSF was added, homogenized on ice, and homogenized results were identified by trypan

blue staining. The tissue homogenate was centrifuged at 1000 g, 4 °C for 5 min. Supernatant was centrifuged at 11000 g, 4 °C for 10 minutes. Supernatant is cytoplasmic protein from which mitochondria are removed, and precipitate is mitochondria isolated. Precipitation Mitochondria are lysed using mitochondrial lysate to obtain mitochondrial protein.

NRCMS mitochondrial proteins were prepared using Cell Mitochondria Isolation Kit (C3601, Beyotime, China). Cells were washed with PBS at room temperature and digested with 0.25 % trypsin EDTA solution, 200 g, centrifuged at room temperature for 5 min to collect cells, and re-suspended by adding mitochondrial isolation reagent, homogenized after 10 min in an ice bath. The cell homogenate was centrifuged at 600 g, 4 °C for 5 minutes. The subsequent steps are identical to those of tissue mitochondrial protein extraction.

***Immunoblotting assay***

As we described previously [5], the cardiac tissue or cultured cells were lysed using RIPA lysis buffer with protease inhibitors and phosphatase inhibitors (Sellck, Shanghai, China). Equal amounts of protein were loaded into SDS-PAGE gel and transferred to PVDF membranes (Millipore, USA). Membranes were incubated overnight with primary antibodies at 4 °C. After incubation with HRP-conjugated anti-mouse secondary antibody (dilution 1: 4000, Proteintech) or anti-rabbit antibody (Proteintech, SA00001 -2, 1/2,000) for 1 hour at room temperature, membranes were exposed to enhanced chemiluminescence (ECL) substrate (Thermo Fisher Scientific, 34580). Western blot results were quantified by Image J.

The primary antibodies used in this study were: anti-RAB7 antibody (ab137029) (1/1000), anti-β-Tubulin antibody (zsbio, TA-10) (1/5000), anti-Bax antibody (Proteintech, 50599-2-Ig) (1/1000), anti-Cleaved caspase-3 antibody (CST, #9664S) (1/1000), anti-PINK1 antibody (Novus, BC100-494) (1/1000), anti-PARKIN antibody (CST, #2132) (1/1000), anti-FUNDC1 antibody (Avivasysbio, OASG02855) (1/1000), anti-SQSTM1/p62 antibody (CST, #39749S) (1/1000), anti-LC3 antibody (Sigma-

Aldrich, L7543) (1/2000), anti-VDAC1-antibody (Proteintech, 55259-1-AP) (1/1000), anti-TUFM-antibody (ABclonal, RK05717) (1/1000).

***Immunoprecipitation assays***

Immunoprecipitation assays were performed as we described previously [1]. Briefly, HEK293T or NRCMS with indicated treatments were lysed with IP lysis buffer (20 mM Tris-HCl, pH 7.4; 150 mM NaCl; 1 mM EDTA; and 1% NP-40) containing protease inhibitor cocktail from Sigma (P8340, St. Louis, MO). After centrifugation at 4 °C, the supernatants were incubated with protein A/G beads (Thermo Fisher, Massachusetts, USA) and the indicated anti-tag antibodies or primary antibodies overnight at 4 °C. For endogenous immunoprecipitation assays, cells were incubated with the indicated primary antibody targeting endogenous proteins. The beads were then washed 3 times with IP lysis buffer. After last wash, interacting proteins were eluted with 1 × SDS loading buffer by boiling at 95 °C for 10 min. Finally, western blotting was performed as mentioned above.

The following immunoprecipitation antibodies were used: anti-RAB7 antibody (dilution 1: 50 for Co-IP, 1: 1000 for Western blot, CST# 95746, Boston, USA), anti-TUFM-antibody (dilution 1: 50 for Co-IP, 1: 1000 for Western blot, Abclonal, Boston, USA), anti-Flag-antibody (dilution 1: 50 for Co-IP, 1: 1000 for Western blot, lot# 2064, Dia-an Biotech, Wuhan, China), anti-HA-antibody (dilution 1: 50 for Co-IP, 1: 1000 for Western blot, HuaAn biotechnology # ET1611-49, Zhejiang, China).

***Immunoprecipitation-mass spectrometry (IP-MS) analysis***

The Immunoprecipitation was performed as described above. Liquid chromatography with tandem mass spectrometry (LC-MS/MS) analysis was performed by the Aksomics (ShangHai, China). Briefly, the IP lysate was blocked by N-ethylmaleimide (NEM, 04269, Sigma-Aldrich, USA) and labelled with biotin-maleimide (B1267, Sigma-Aldrich, USA). Biotinylated proteins were then trypsinized using trypsin with a ratio of 1:50 to protein for 16 h and purified with Pierce™

NeutrAvidin™ Plus UltraLink™ Resin (53151, ThermoFisher, USA) for LC-MS/MS analysis. Peptides were separated by the NanoLC 1D Plus system. Eluting peptide cations were ionized using a nanospray and analyzed using an LTQ-Orbitrap XL mass spectrometer (Thermo Fisher, San Jose, USA). The MS/MS spectra were searched against the UniProt rat proteome database using SequestHT.

## Supplemental references


1. Zheng D, Liu Z, Zhou Y, Hou N, Yan W, Qin Y, et al. Urolithin B, a gut microbiota metabolite, protects against myocardial ischemia/reperfusion injury via p62/Keap1/Nrf2 signaling pathway. Pharmacol Res. 2020; 153: 104655.
2. Wu X, Qin Y, Zhu X, Liu D, Chen F, Xu S, et al. Corrigendum to increased expression of DRAM1 confers myocardial protection against ischemia via restoring autophagy flux. J Mol Cell Cardiol. 2019; 127: 277.
3. Wu X, Qin Y, Zhu X, Liu D, Chen F, Xu S, et al. Increased expression of DRAM1 confers myocardial protection against ischemia via restoring autophagy flux. J Mol Cell Cardiol. 2018; 124: 70-82.
4. Li J, Song Y, Jin JY, Li GH, Guo YZ, Yi HY, et al. CD226 deletion improves post-infarction healing via modulating macrophage polarization in mice. Theranostics. 2020; 10: 2422-35.
5. Wu X, Huang J, Tang J, Sun Y, Zhao G, Yan C, et al. Isoginkgetin, a bioactive constituent from Ginkgo Biloba, protects against obesity-induced cardiomyopathy via enhancing Nrf2/ARE signaling. Redox Biol. 2022; 57: 102485.
6. Tong M, Mukai R, Mareedu S, Zhai P, Oka SI, Huang CY, et al. Distinct Roles of DRP1 in Conventional and Alternative Mitophagy in Obesity Cardiomyopathy. Circ Res. 2023; 133: 6-21.

## Supplemental Figures

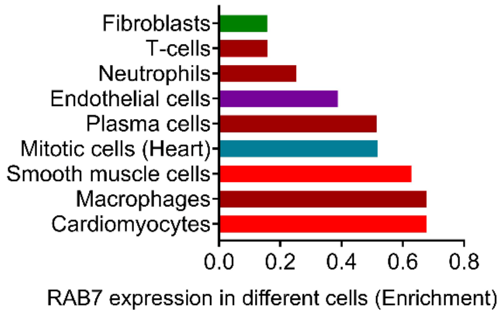


**Figure S1. RAB7 expression in various types of hearts cells.**

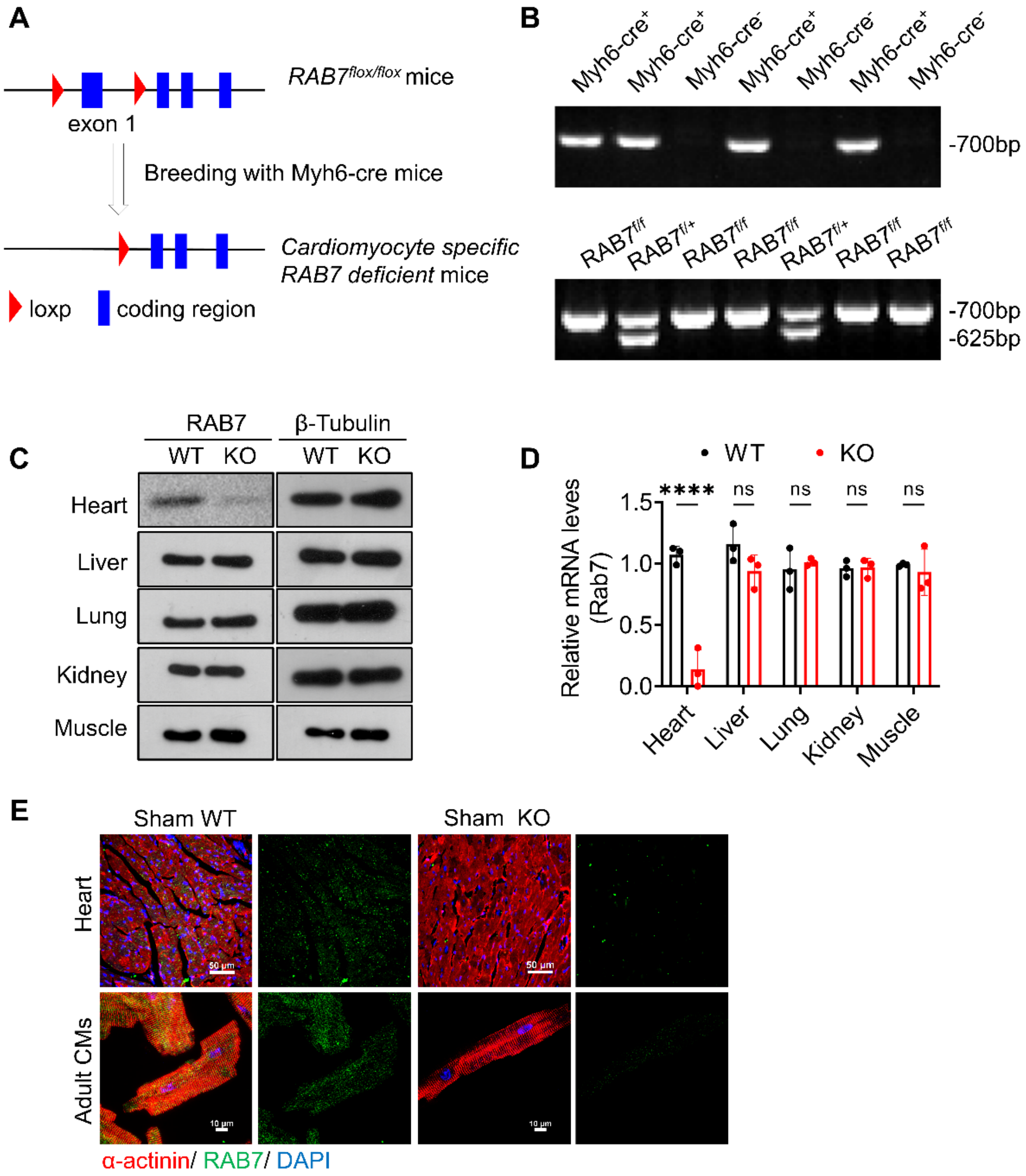


**Figure S2. Generation of myocardium-specific knockout mice. A,** Myh6-cre crossed with RAB7$^{flox/flox}$ to generate Myh6-tcre; RAB7$^{flox/flox}$ (cKO) mice. **B,** Genotype PCR results of RAB7$^{flox/flox}$, RAB7$^{flox/+}$ and Myh6-cre$^{+}$ mouse. **C, D,** RAB7 protein and mRNA levels in heart, liver, lung, kidney and muscle tissues from WT and cKO mice measured by western blotting and qRCR. **E,** RAB7 immunofluorescence confirmed the complete RAB7 deletion in heart and adult cardiomyocytes. Scale bar = 50 μm (above), 10 μm (below).

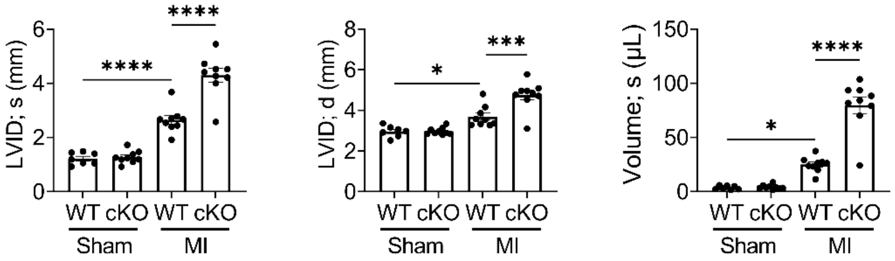


**Figure S3. RAB7 cKO mice elevates left ventricular diameter.** Echocardiographic parameters recorded in (Figure 2A) (n = 7–9). LVID; s, systolic left ventricular internal dimension; LVID; d, diastolic left ventricular internal dimension; Volume; s, left ventricular end-systolic volume. Data are presented as the mean ± SEM. *P* values were calculated by two-way ANOVA followed by Tukey's test. $^{*}P < 0.05$, $^{***}P < 0.001$, $^{****}P < 0.0001$.

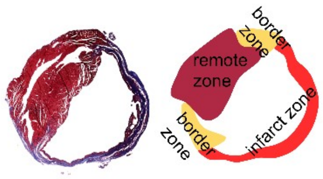


**Figure S4. The diagram of the infarct zone, border zone and remote zone.**

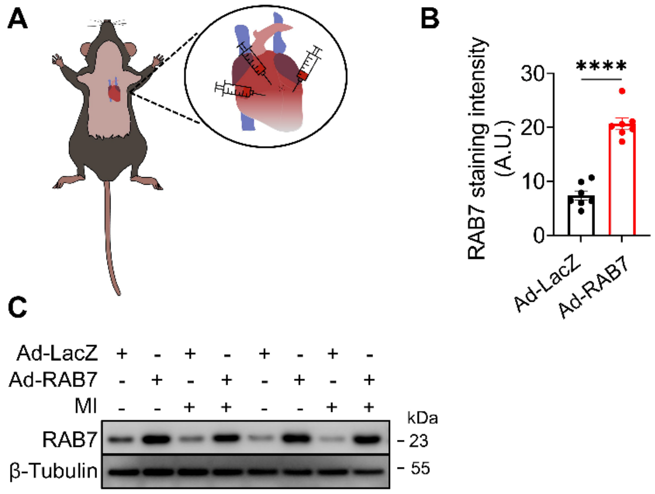


**Figure S5. transfection with LacZ and RAB7 adenovirus. A,** Pattern of LacZ and RAB7 adenovirus injection in situ myocardium. **B,** Quantification of immunofluorescent staining of RAB7 in MI heart sections (Figure 3A) (n=7). **C,** Immunoblot images of RAB7 expression in the border zone of different groups mice. Data are presented as the mean ± SEM. *P* values were calculated by unpaired two-tailed Student's t-test. $^{****}P < 0.0001$.

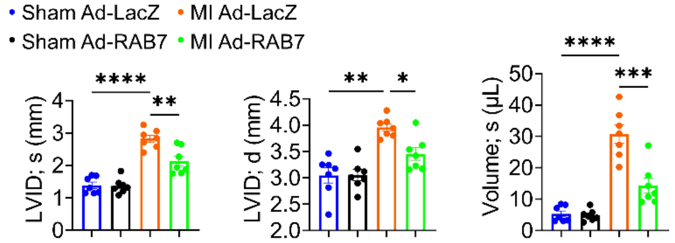


**Figure S6. Overexpression of RAB7 improves cardiac dysfunction.** Echocardiographic parameters recorded in (Figure 3B) (n = 7–9). Data are presented as the mean ± SEM. *P* values were calculated by two-way ANOVA followed by Tukey's test. $^{*}P < 0.05$, $^{**}P < 0.01$, $^{***}P < 0.001$, $^{****}P < 0.0001$.

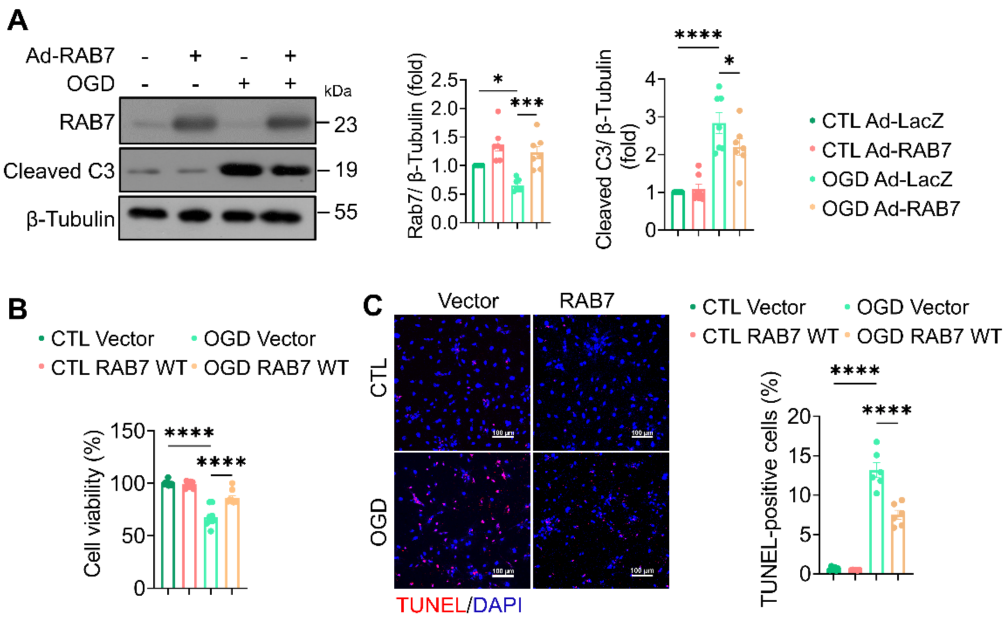


**Figure S7. RAB7 overexpression decreases apoptosis in cardiomyocytes.** NRCMS were transfected with LacZ adenovirus, RAB7 adenovirus, Vector or RAB7 plasmid for 24 h, and then exposed to OGD for 3 h. **A,** Western blot and quantitation of RAB7 and Cleaved caspase 3 protein expression in different groups post-hypoxia. (n = 6). **B,** ccK-8 cell viability assay of NRCMS. (n = 9). **C,** TUNEL staining and quantification to assay cell death (n = 6). Scale bar = 100 μm. Data are presented as the mean ± SEM. *P* values were calculated by two-way ANOVA followed by Tukey's test. $^{*}P < 0.05$, $^{**}P < 0.01$, $^{***}P < 0.001$, $^{****}P < 0.0001$.

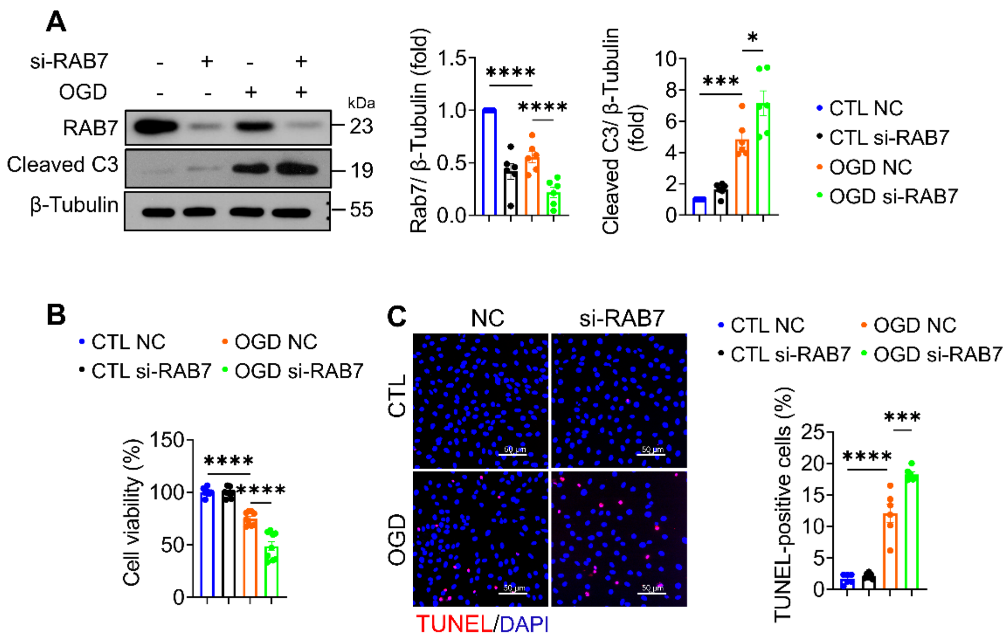


**Figure S8. Knockdown RAB7 increases apoptosis in cardiomyocytes.** NRCMS were transfected with NC siRNA or RAB7 siRNA for 24 h, and then exposed to OGD for 3 h. **A,** Western blot and quantitation of RAB7 and Cleaved caspase 3 protein expression in different groups post-hypoxia. (n = 6). **B,** ccK-8 cell viability assay of NRCMS (n = 9). **C,** TUNEL staining and quantification to assay cell death (n = 6). Scale bar = 50 μm. Data are presented as the mean ± SEM. *P* values were calculated by two-way ANOVA followed by Tukey's test. $^{*}P < 0.05$, $^{**}P < 0.01$, $^{***}P < 0.001$, $^{****}P < 0.0001$.

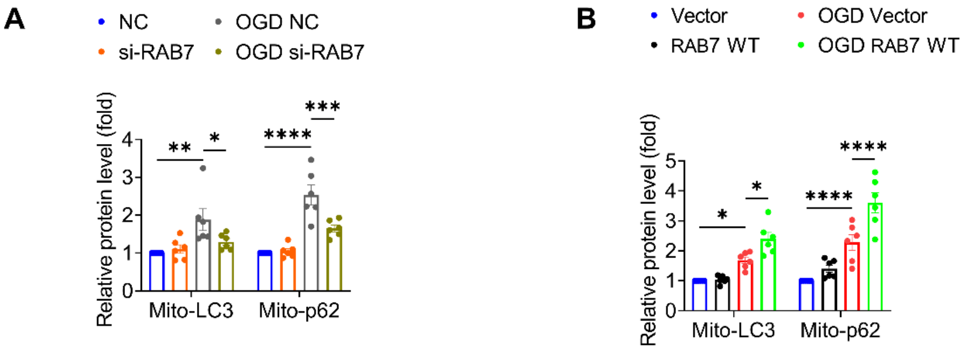


**Figure S9. RAB7 deletion reduces early mitophagy after OGD. A-B**, Quantitative analysis of LC3 and p62 protein levels in cytoplasmic and mitochondrial fraction

prepared in (Figure 5M-N). (n = 6). Data are presented as the mean ± SEM. *P* values were calculated by two-way ANOVA followed by Tukey's test. $^{*}P < 0.05$, $^{**}P < 0.01$, $^{***}P < 0.001$, $^{****}P < 0.0001$.

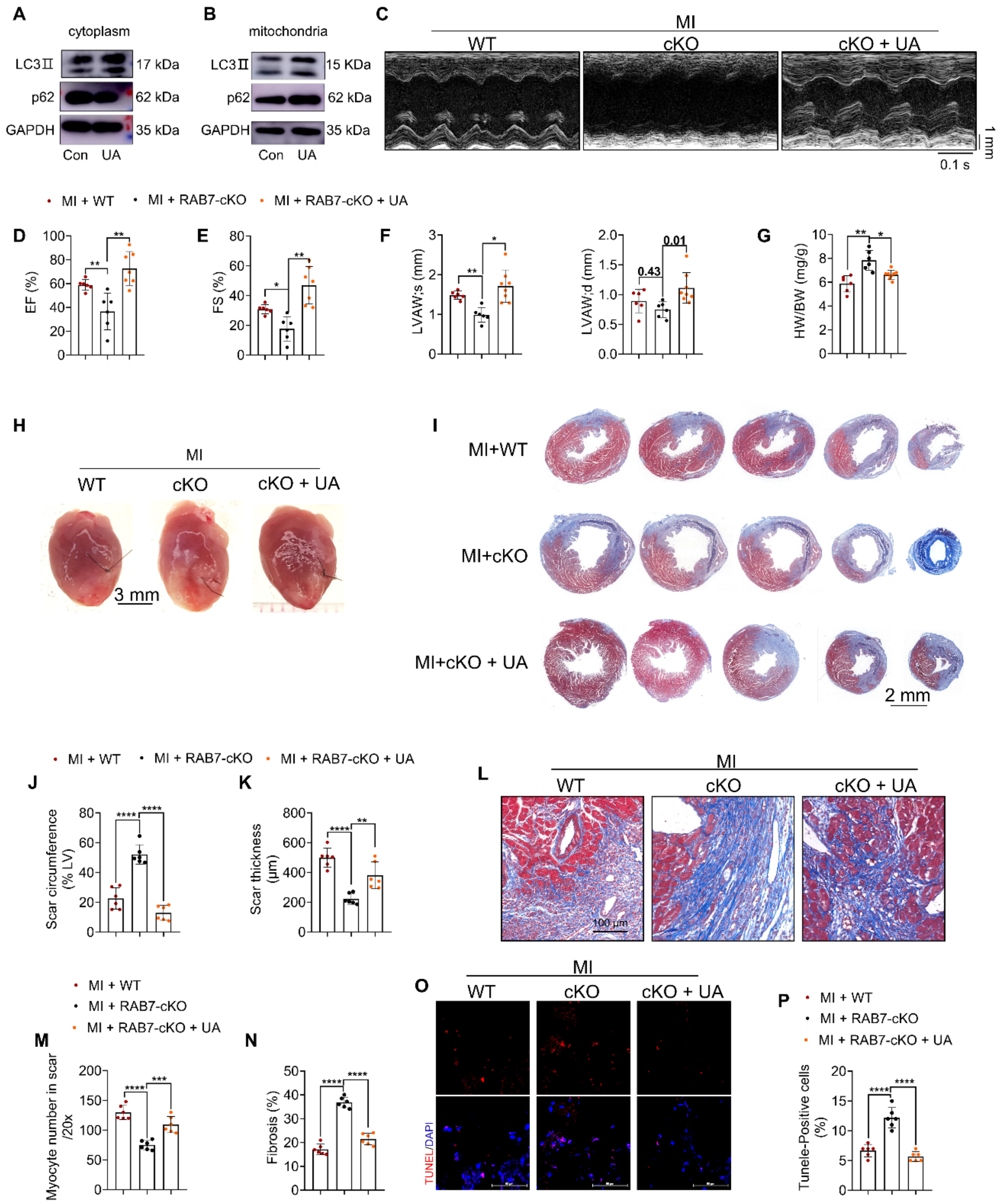


**Figure S10. Reactivation mitophagy by using Urolithin A (UA) improves cardiac function of RAB7 deletion mice post MI.** UA (30 mg/kg/day) was administrated into mice to induce mitophagy. **A-B,** The protein expression of LC3II and p62 in the

cytoplasm fraction (A) and the mitochondrial fraction (B) was detected by Western blot. As an activator of mitophagy, UA (30 mg/kg/d) was administrated to RAB7-cKO mice post MI for two weeks. The cardiac function of mice was measured by M-mode echocardiography. **C,** Representative echocardiographic images from mice in each group. **D-F,** Echocardiographic parameters recorded in (n = 6 – 8) **G,** HW/BW ratio in each group (n = 6 - 8). **H,** Representative images of hearts from each group at day 14 following MI. Scale bar = 3 mm. **I,** Representative five sections of Masson's trichrome staining are illustrated. Scale bar = 2 mm. **J,** Quantification of scar circumference (n = 6). **K,** Quantification of scar thickness (n = 6). **L,** Representative image of scar at higher magnification. Scale bar = 100 μm. **M,** Quantification of viable myocardium in scar areas. Viable myocardium stained red (n = 6). **N,** Ratio of collagen volume fraction in the border zone were quantitatively estimated in 5 randomly chosen higher magnification fields each section (n = 6). **O-P,** Images and quantitative analysis of TUNEL staining in the scar tissue isolated from mice at day 3 following MI (n = 6). Data are presented as the mean ± SEM. *P* values were calculated by two-way ANOVA followed by Tukey's test. $^{*}P < 0.05$, $^{**}P < 0.01$, $^{***}P < 0.001$, $^{****}P < 0.0001$.

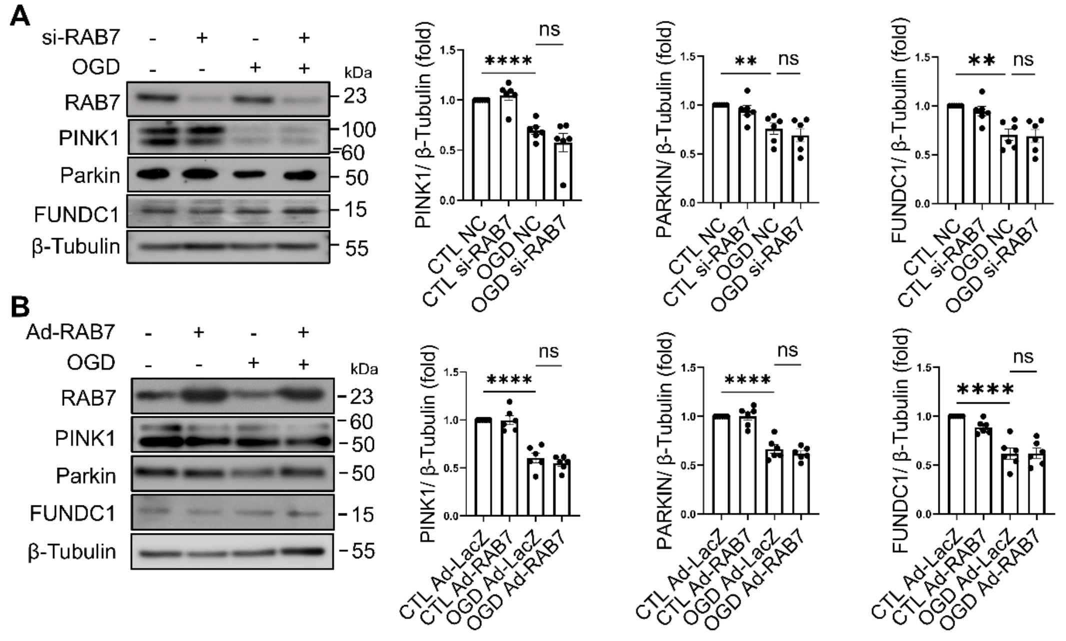


**Figure S11. RAB7 deletion does not alter the expression of PINK1/PARKIN protein in classical mitophagy pathway.** NRCMS were transfected with NC siRNA, RAB7 siRNA, LacZ adenovirus or RAB7 adenovirus for 24 h, and then exposed to OGD for 24 h. **A-B,** Immunoblot analysis of PINK1, Parkin and FUNDC1 protein levels in NRCMs (n = 6). Data are presented as the mean ± SEM. *P* values were calculated by two-way ANOVA followed by Tukey's test. $^{**}P < 0.01$, $^{****}P < 0.0001$.

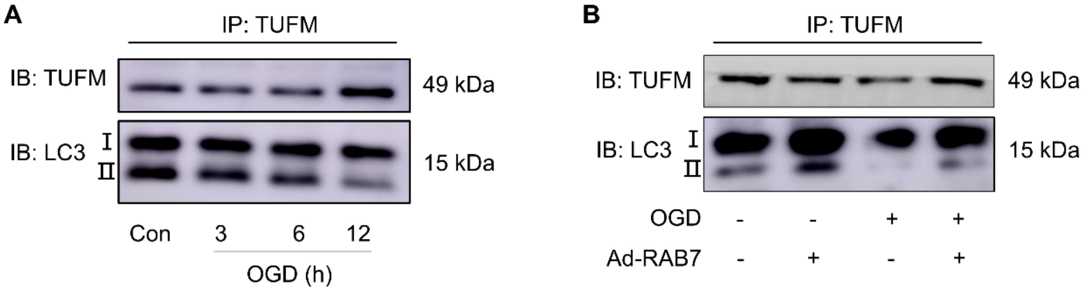


**Figure S12. The interaction of TUFM with LC3 in cardiomyocytes under OGD stress was detected. A,** The interaction of TUFM with LC with OGD stimulation at different time points. **B,** The interaction was detected with RAB7 overexpression.

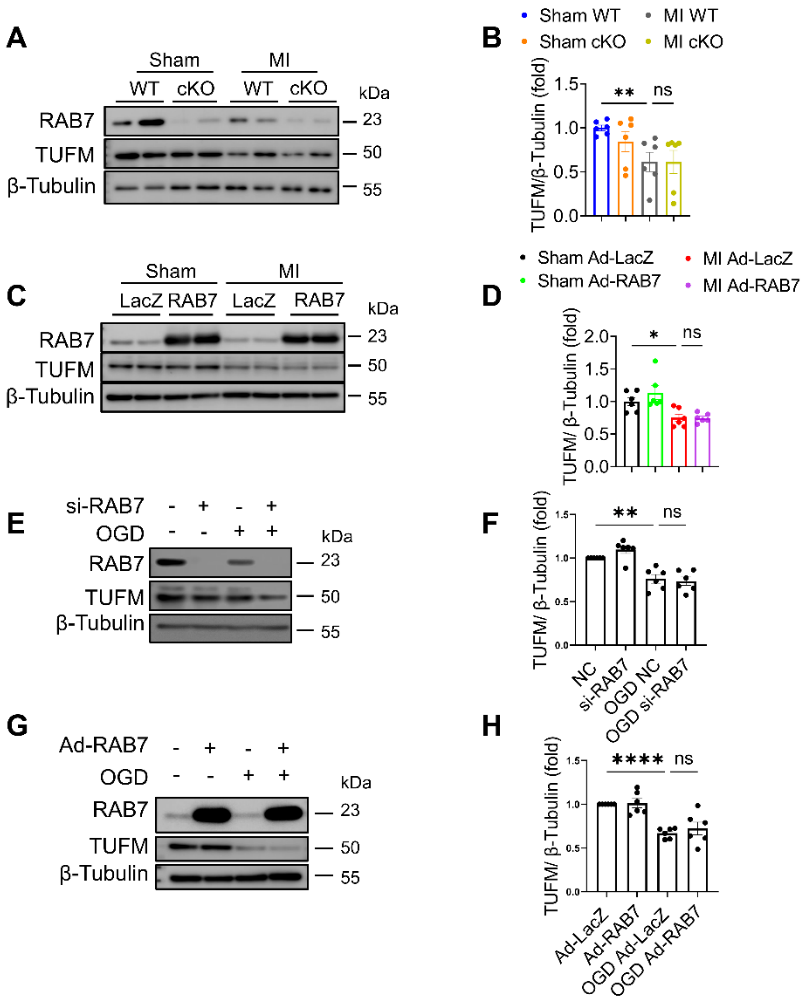


**Figure S13. Deletion or overexpression of RAB7 does not affect TUFM protein expression levels. A, C,** Immunoblot images of TUFM expression in the border zone in the indicated groups at day 14 post-MI as well as in the sham control. **B, D,** Quantitative analysis TUFM protein levels (n = 6). **E, G,** Immunoblot images of TUFM expression of NRCMS post-hypoxia. **F, H,** Quantitative analysis TUFM protein levels (n = 6). Data are presented as the mean ± SEM. *P* values were calculated by two-way ANOVA followed by Tukey's test. $^{*}P < 0.05$, $^{**}P < 0.01$, $^{****}P < 0.0001$.

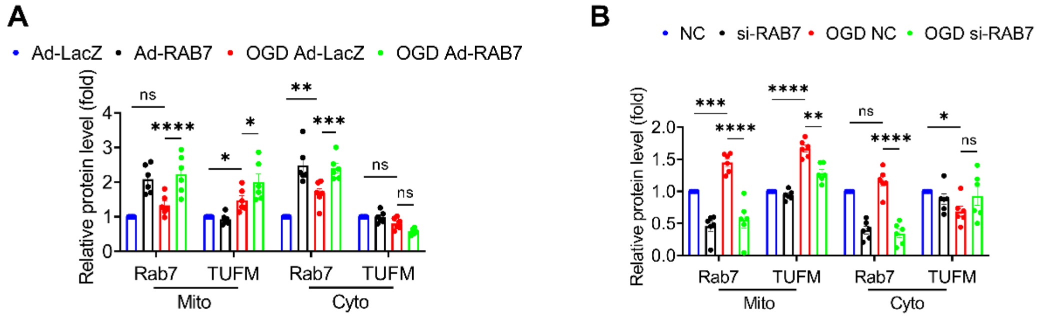


**Figure S14. RAB7 regulates the translocation of TUFM in the cytoplasm. A-B,** Quantitative analysis of RAB7 and TUFM protein levels in cytoplasmic and mitochondrial fraction prepared in (Figure 7F-G) (n = 6). Data are presented as the mean ± SEM. *P* values were calculated by two-way ANOVA followed by Tukey's test. $^{*}P < 0.05$, $^{**}P < 0.01$, $^{***}P < 0.001$, $^{****}P < 0.0001$.

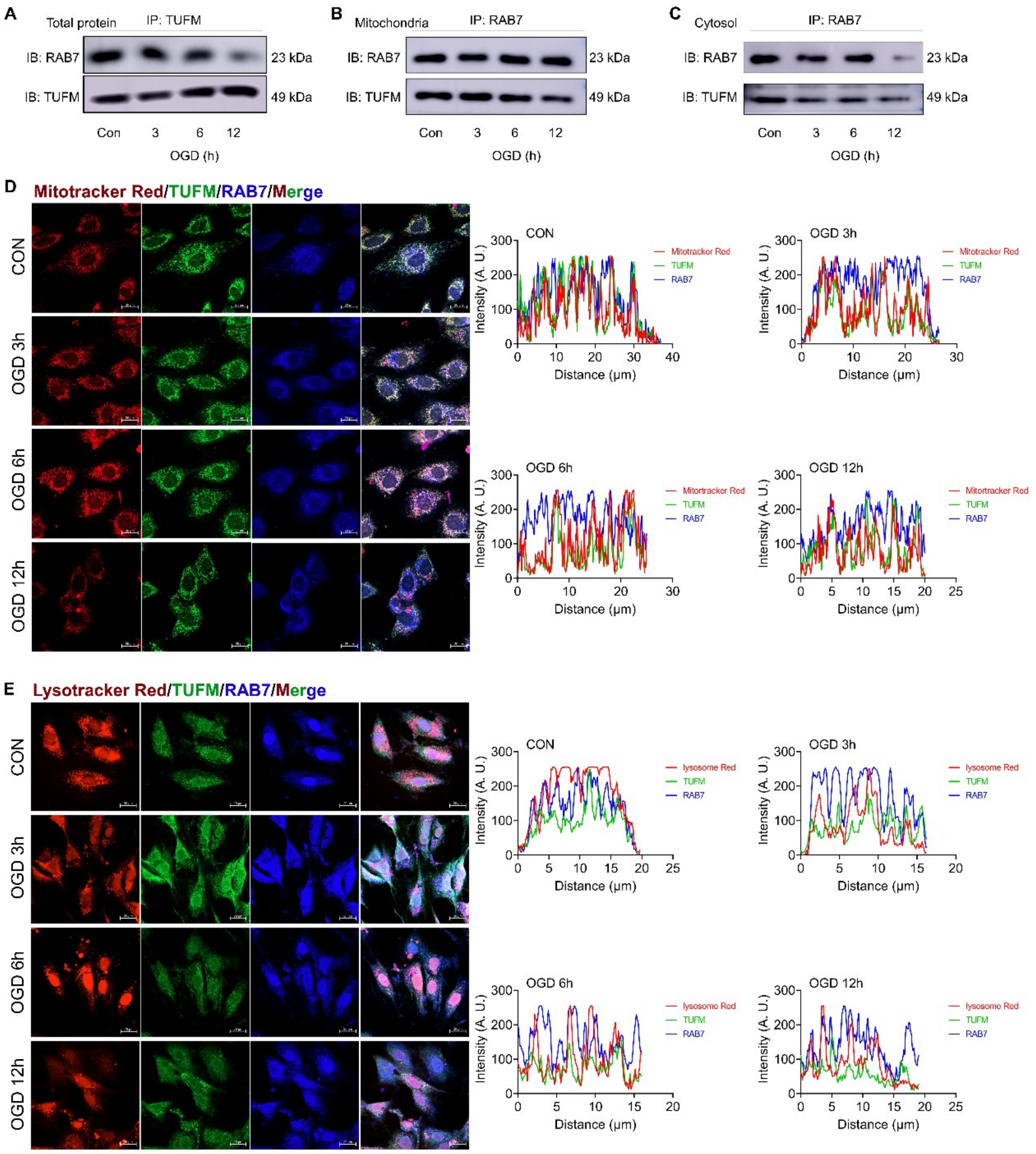


**Figure S15.** The interaction of RAB7 with TUFM and the subcellular localization of RAB7-TUFM complex in cardiomyocytes with OGD stimulation were assayed. **A,** TUFM was immunoprecipitated by antibodies and the interaction with RAB7 was detected by co-IP assay at different time points with OGD stress. **B and C,** the interaction of RAB7 with TUFM in the mitochondrial or cytoplasmic fraction was detected by co-IP at different time points with OGD stimulation. **D,** the localization of RAB7-TUFM complex in the mitochondrial fraction under OGD condition at different time points. **E,** the localization of RAB7-TUFM complex in the lyososomal fraction under OGD condition at different time points. The colocalization of RAB7 and TUFM

interaction in mitochondrial or lysosomal fraction were quantified by using Image J.

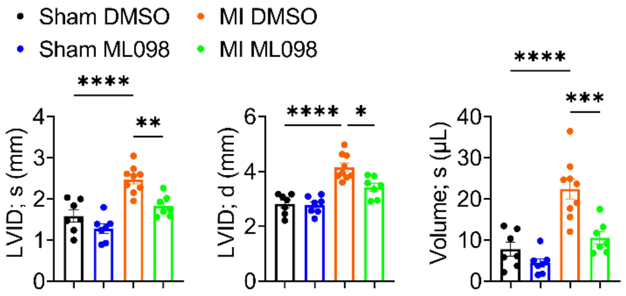


**Figure S16. Pharmacological activation of RAB7 reduces myocardial damage.** Echocardiographic parameters recorded in (Figure 8A) (n = 7–9). Data are presented as the mean ± SEM. *P* values were calculated by two-way ANOVA followed by Tukey's test. $^{*}P < 0.05$, $^{**}P < 0.01$, $^{***}P < 0.001$, $^{****}P < 0.0001$.

**Table 1: Echocardiographic results of WT mice and cKO mice mice at baseline.**

| | Units | WT | cKO |
|---|---|---|---|
| **N** | | 6 | 8 |
| **Heart Rate** | BPM | 566.3± 15.78 | 583.3± 22.32 (*p*=0.5727) |
| **Diameter; s** | mm | 1.184± 0.08955 | 1.377± 0.08375 (*p*=0.1461) |
| **Diameter; d** | mm | 2.898± 0.09478 | 2.982± 0.06456 (*p*=0.4623) |
| **Volume; s** | µL | 3.47± 0.6923 | 5.153± 0.8158 (*p*=0.1589) |
| **Volume; d** | µL | 32.51± 2.518 | 34.7± 1.900 (*p*=0.4907) |
| **Stroke Volume** | µL | 29.04± 2.262 | 29.55± 1.735 (*p*=0.8578) |
| **EF** | % | 89.42± 1.845 | 85.16± 2.180 (*p*=0.1793) |
| **FS** | % | 59.2± 2.611 | 53.83± 2.624 (*p*=0.1815) |
| **LVAW;s** | mm | 1.374±0.04245 | 1.353±0.04895 (*p*=0.7640) |
| **LVAW;d** | mm | 0.7701±0.06077 | 0.7121±0.05830 (*p*=0.5110) |

Statistical analysis was evaluated by two-tailed unpaired t-test. Data represent the mean ± SEM.

**Table 2: Cardiac function of mice post MI for 14 days was evaluated by echocardiographic analysis.**

| | Sham | | MI | |
|---|---|---|---|---|
| | **WT** | **cKO** | **WT** | **cKO** |
| **N** | 7 | 9 | 9 | 9 |
| **Heart Rate** | 561.5±14.18 | 549.3±25.23 (*p*=0.9999) | 546.8±30.25 | 543.9±35.19 (*p*=0.9998) |
| **Diameter; s** | 1.221±0.08425 | 1.276±0.07644 (*p*=0.9962) | 2.646±0.1552 | 4.300±0.2546 (*p*< 0.0001) |
| **Diameter; d** | 2.964±0.1040 | 2.973±0.05838 (*p*=0.9984) | 3.691±0.1719 | 4.751±0.2364 (*p*=0.0005) |
| **Volume; s** | 3.760±0.6529 | 4.255±0.6828 (*p*=0.9995) | 25.04±2.332 | 79.87±7.664 (*p*< 0.0001) |
| **Volume; d** | 34.47±2.892 | 34.45±1.711 (*p*> 0.9999) | 48.72±5.198 | 101.3±8.399 (*p*< 0.0001) |
| **Stroke Volume** | 30.71±2.538 | 30.19±1.381 (*p*=0.9996) | 24.80±4.088 | 21.46±1.700 (*p*=0.7200) |
| **EF** | 89.24±1.571 | 87.82±1.615 (*p*=0.9816) | 47.71±2.074 | 21.60±2.511 (*p*< 0.0001) |
| **FS** | 58.90±2.227 | 57.18±2.193 (*p*=0.9782) | 23.74±1.238 | 9.890±1.188 (*p*< 0.0001) |
| **LVAW;s** | 1.386±0.03782 | 1.355±0.04322 (*p*=0.9910) | 1.060±0.06826 | 0.6219±0.05908 (*p*< 0.0001) |
| **LVAW;d** | 0.7673±0.05143 | 0.7108±0.05144 (*p*=0.9782) | 0.7248±0.03869 | 0.4975±0.01777 (*p*=0.0019) |

Two-way ANOVA analysis followed by Tukey's multiple comparison test was conducted to compare the cardiac functions of mice between WT and cKO in sham or MI group. Data represent the mean ± SEM.

**Table 3: Cardiac function of mice post MI for 14 days was evaluated by echocardiographic analysis.**

| | Sham | | MI | |
|---|---|---|---|---|
| | **Ad-LacZ** | **Ad-RAB7** | **Ad-LacZ** | **Ad-RAB7** |
| **N** | 7 | 7 | 7 | 7 |
| **Heart Rate** | 604.9±13.95 | 595.9±12.91 (*p*=0.9865) | 558.9±27.26 | 603.4±20.46 (*p*=0.4691) |
| **Diameter; s** | 1.389±0.09732 | 1.376±0.08953 (*p*=0.9990) | 2.825±0.1096 | 2.134±0.1547 (*p*=0.0017) |
| **Diameter; d** | 3.046±0.1444 | 3.050±0.1058 (*p*=0.9995) | 3.955±0.07131 | 3.454±0.1202 (*p*=0.0463) |
| **Volume; s** | 5.310±0.9449 | 4.845±0.6866 (*p*=0.9976) | 30.74±2.884 | 14.25±2.415 (*p*= 0.0004) |
| **Volume; d** | 37.17±3.869 | 37.53±3.667 (*p*> 0.9999) | 59.85±4.021 | 43.27±2.801 (*p*= 0.0216) |
| **Stroke Volume** | 31.86±3.372 | 32.41±2.873 (*p*=0.9991) | 29.12±2.297 | 33.52±3.264 (*p*=0.3102) |
| **EF** | 80.19±1.909 | 82.22±1.268 (*p*=0.9743) | 48.85±2.650 | 70±2.634 (*p*< 0.0001) |
| **FS** | 54.25±2.559 | 55.59±1.585 (*p*=0.9531) | 24.31±1.577 | 40.59±1.759 (*p*< 0.0001) |
| **LVAW;s** | 1.363±0.06193 | 1.514±0.07187 (*p*=0.2499) | 0.6280±0.04297 | 1.003±0.07112 (*p*= 0.0180) |
| **LVAW;d** | 0.7441±0.04312 | 0.8789±0.08326 (*p*=0.1045) | 0.4253±0.02635 | 0.6413±0.04471 (*p*=0.0359) |

Two-way ANOVA analysis followed by Tukey's multiple comparison test was conducted to compare the cardiac functions of mice between Ad-LacZ and Ad-RAB7 in sham or MI group. Data represent the mean ± SEM.

**Table 4: Echocardiographic analysis of cardiac function of mice post MI for 14 days.**

| | Units | MI+WT | MI+cKO | MI+cKO+UA |
|---|---|---|---|---|
| **N** | | 6 | 6 | 8 |
| **Heart Rate** | BPM | 432.3± 35.59 | 542.4±22.51 (*p*=0.0230) | 462.8±20.72 (*p*=0.0844) |
| **Diameter; s** | mm | 2.807± 0.09016 | 3.067± 0.2482 (*p*=0.5480) | 1.557± 0.1732 (*p*<0.0001) |
| **Diameter; d** | mm | 4.042± 0.1221 | 3.713± 0.5626 (*p*=0.2297) | 2.929± 0.4104 (*p*=0.1451) |
| **Volume; s** | µL | 29.85± 2.698 | 38.99± 7.122 (*p*=0.3003) | 7.891± 2.176 (*p*=0.0001) |
| **Volume; d** | µL | 72.28± 5.338 | 60.39± 8.720 (*p*=0.3658) | 33.99± 4.191 (*p*=0.0119) |
| **Stroke Volume** | µL | 42.42± 3.162 | 21.41± 4.863 (*p*=0.0017) | 26.10± 2.563 (*p*=0.5750) |
| **EF** | % | 58.86± 1.853 | 36.58± 6.289 (*p*=0.00138) | 72.65± 5.332 (*p*=0.0002) |
| **FS** | % | 30.85± 1.245 | 17.67± 33.349 (*p*=0.0466) | 46.90± 4.758 (*p*<0.0001) |
| **LVAW;s** | mm | 1.485±0.04205 | 0.9887± 0.07625 (*p*=0.0155) | 1.713± 0.1436 (*p*=0.0004) |
| **LVAW;d** | mm | 0.8906± 0.07978 | 0.7472±0.05354 (*p*=0.4359) | 1.112± 0.09060 (*p*=0.0095) |

Two-way ANOVA analysis followed by Tukey's multiple comparison test was conducted to compare the cardiac functions of mice between cKO and WT or between UA treated cKO and cKO mice in MI condition. Data represent the mean ± SEM.

**Table 5: Cardiac function of mice post MI for 14 days was evaluated by echocardiographic analysis.**

| | Sham | | MI | |
|---|---|---|---|---|
| | **DMSO** | **ML-098** | **DMSO** | **ML-098** |
| **N** | 7 | 7 | 9 | 7 |
| **Heart Rate** | 513.8±32.54 | 605.4±25.62 (*p*=0.1035) | 563.0±27.43 | 602.0±18.57 (*p*=0.9220) |
| **Diameter; s** | 1.585±0.1477 | 1.281±0.1167 (*p*=0.3048) | 2.477±0.1094 | 1.830±0.09693 (*p*= 0.0017) |
| **Diameter; d** | 2.824±0.1428 | 2.773±0.1218 (*p*=0.9902) | 4.156±0.1567 | 3.429±0.1460 (*p*=0.0232) |
| **Volume; s** | 7.778±1.675 | 4.456±1.041 (*p*=0.5586) | 22.45±2.437 | 10.54±1.462 (*p*= 0.0001) |
| **Volume; d** | 41.06±3.910 | 35.22±3.485 (*p*= 0.0.5760) | 40.44±2.762 | 31.55±2.900 (*p*= 0.0712) |
| **Stroke Volume** | 33.28±2.503 | 30.77±2.625 (*p*=0.8145) | 17.98±0.8566 | 21.01±2.503 (*p*=0.8690) |
| **EF** | 82.22±2.760 | 88.05±1.892 (*p*=0.5093) | 45.53±2.537 | 66.30±3.847 (*p*= 0.0002) |
| **FS** | 50.74±3.060 | 57.52±2.632 (*p*=0.3058) | 22.04±1.459 | 35.84±2.859 (*p*= 0.0058) |
| **LVAW;s** | 1.532±0.1115 | 1.607±0.1067 (*p*=0.9782) | 1.011±0.07242 | 1.232±0.1891 (*p*= 0.4886) |
| **LVAW;d** | 0.9225±0.1088 | 0.9809±0.09729 (*p*=0.9801) | 0.8182±0.06880 | 0.8447±0.1369 (*p*=0.9226) |

Two-way ANOVA analysis followed by Tukey's multiple comparison test was conducted to compare the cardiac functions of mice between DMSO and ML-098 in sham or MI group. Data represent the mean ± SEM.

**Table6: Primer sequence used in Real time PCR.**

| Gene | | Primer Sequences (5'to3') |
|---|---|---|
| RAB7 | Forward | GGC AAG GCA AGC ACT ACT GT |
| | Reverse | CAA ACT CAC TCA CTC CTA AAT GG |
| 36B4 | Forward | CAG CAA GTG GGA AGG TGT AAT CC |
| | Reverse | CCC ATT CTA TCA TCA ACG GGT ACA A |
